# Asymmetric Total Synthesis of Leptosphin C

**DOI:** 10.1021/acs.orglett.5c03236

**Published:** 2025-08-26

**Authors:** Lantian Sun, Chenshan Lian, Jingyi Zhao, Xiaole Chen, Wei Han, Zigang Li, Chi-Sing Lee

**Affiliations:** † Department of Chemistry, 26679 Hong Kong Baptist University, Waterloo Road, Kowloon Tong, Hong Kong SAR 999077, China; ‡ State Key Laboratory of Chemical Oncogenomics, School of Chemical Biology and Biotechnology, 429362 Peking University Shenzhen Graduate School, Shenzhen University Town, Xili, Shenzhen 518055, China

## Abstract

A concise enantioselective total synthesis of leptosphine C (**1**) has been achieved. Our synthetic strategy features an unprecedented diastereo-/enantioselective Hajos–Parrish–Eder–Sauer–Wiechert (HPESW) reaction for construction of the AB ring system with continuous stereogenic centers (76% yield, 90% ee, dr = 19:1) and an uncommon Pauson–Khand (PK) reaction of geminal dimethyl alkenyl side chains for establishing the CD ring system with the quaternary carbon at C14.

## Graphical abstract

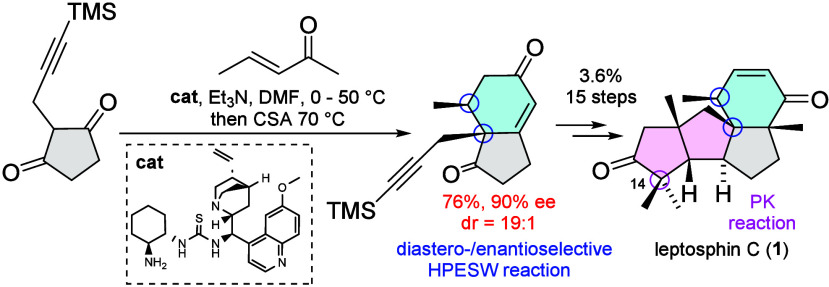

Cyclopianes are a class of diterpenes characterized by a linearly fused *cis*-*anti*-*cis*-5,5,5-tricyclic (BCD) ring system, with a six-membered (A) ring angularly fused into the B ring, forming a highly congested tetracyclic ABCD-ring skeleton (Figure 1). Conidiogenol and conidiogenone were the first two members of cyclopianes isolated from the extracts of the fermentation broth of *Penicillium cyclopium* in 2002, and they exhibited potent conidiation-inducing activity.^1^ Other congeners including conidiogenol B–D,^2,5^ conidiogenone B–L,^2–5^ and three hydroxy conidiogenones^6^ were all isolated from the *Penicillium* genus, except leptosphin C, which was isolated from an endophytic fungus *Leptosphaeria* sp. XL026.^7^ These cyclopiane diterpenoids show a variety of biological activities such as antibacterial, anti-inflammatory, and anticancer activities. Due to their low natural abundance and their unique array of structural features with diverse biological activities, a considerably large amount of synthetic efforts on these natural products have been reported.^8–18^ In 2016, Tu’s group reported the first total synthesis of conidiogenone, conidiogenol, and conidiogenone B and also corrected their absolute configurations.^13^ As shown in Figure 2A, Tu’s synthetic strategy featured an intramolecular [2 + 2] cycloaddition and semipinacol-type rearrangement for C ring formation and a Grignard addition followed by aldol cyclization to construct the B ring. In 2019, Snyder’s group reported a quaternary-center-guided strategy, which involved Baran’s reductive coupling, that intercepted Heck and reductive Heck reactions to establish the D, B, and A ring, respectively.^14^ Zhai’s synthesis of cyclopiane dispersoids utilized the Van Leusen reaction, the Nicholas/Pauson–Khand (PK) cascade reaction for the construction of the BC ring system, and Danheiser annulation followed by aldol cyclization for A ring formation.^15^ In 2023, Lee’s group reported an elegant trimethylenemethan (TMM) diyl-medicated cycloaddition, in which the BCD ring was established in a single step.^16,17^ Recently, Hao’s group reported their strategy utilizing the PK reaction for the construction of the BC ring. After Sonogashira cross-coupling, the D ring was established through 3,3-rearrangement followed by Nazarov cyclization.^18^

**Figure 1:**
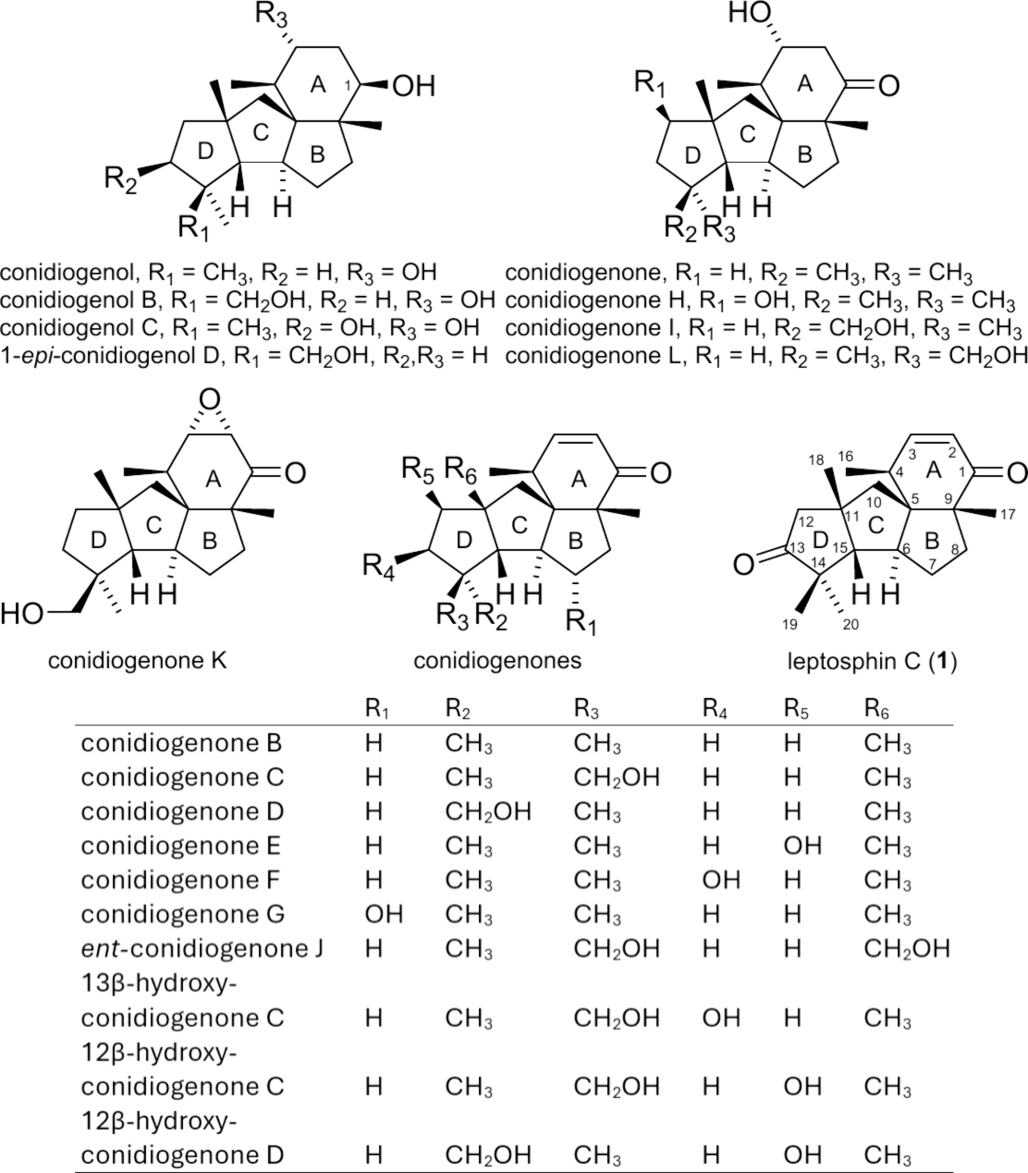
Structures of cyclopiane diterpenoids.

**Figure 2:**
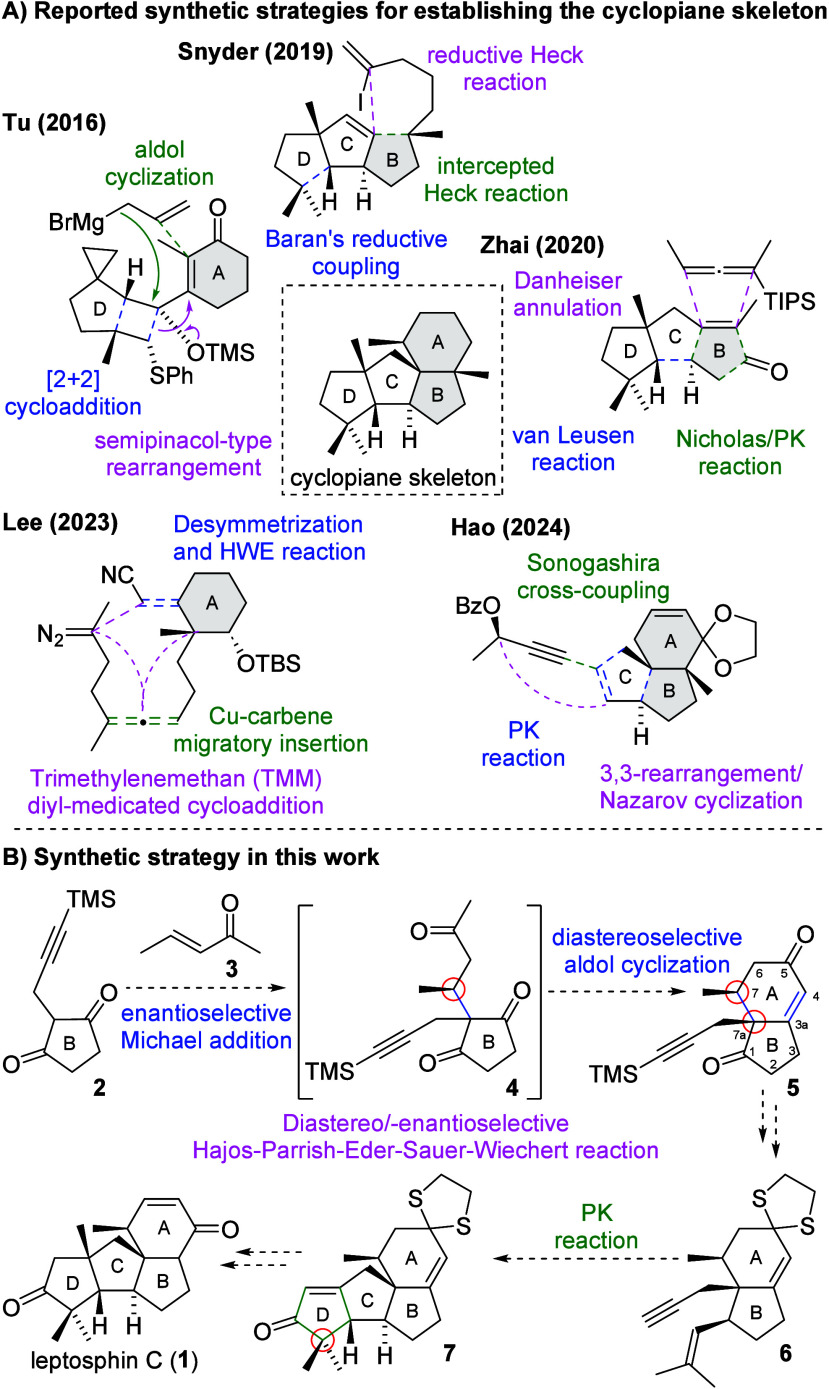
A) Reported synthetic strategies for establishing the cyclopiane skeleton. B) Synthetic strategy in this work.

Our group has been focused on developing an efficient method for the asymmetric synthesis of a 7-substituted Hajos–Parrish–Eder–Sauer–Wiechert (HPESW) ketone, which features a 5,6-bicyclic ring system with continuous stereogenic centers—a common motif in natural products.^19–22^ As shown in Figure 2B, our synthetic strategy involved a diastereo-/enantioselective HPESW reaction between 1,3-diketone **2** and enone **3**, which should provide 7*S*-methyl HPESW ketone **5** in one pot. After installation of the geminal dimethyl alkenyl side chain to the B ring, PK reaction of **6** would establish the tetracyclic core of **7** with the quaternary carbon center in the D ring. Finally, **7** would be further elaborated to leptosphin C (**1**). The major challenge of this strategy is to identify an asymmetric catalytic system capable of facilitating the initial enantioselective Michael addition between 1,3-diketone **2** and enone **3** as well as the succeeding diastereoselective intramolecular aldol cyclization of chiral intermediate **4**. This involves managing potential match or mismatch scenarios between the chirality of the catalyst and **4**. To investigate the feasibility of this strategy, we first studied the effects of the stereogenic center of **4** in the intramolecular aldol cyclization step using DFT calculations. As shown in Scheme 1A, transition state **TS1** is more favorable than transition state **TS2** with an energy difference of 6.36 kcal/mol (all energies are relative to intermediate **4**), due to an eclipsed conformation between the methyl group and the 1,3-diketone moiety in **TS2**. Moreover, **TS1** could lead to the desired product **8a**, which is the thermodynamic product, with an energy difference of 1.24 kcal/mol that is more favorable than that of **8b**. These results indicated that the cyclization product (**8a**), which can lead to the desired 7*S*-methyl HPESW ketone **5**, is more favorable under substrate control, and its diastereoselectivity could possibly be enhanced under thermodynamic conditions.

**Scheme 1:**
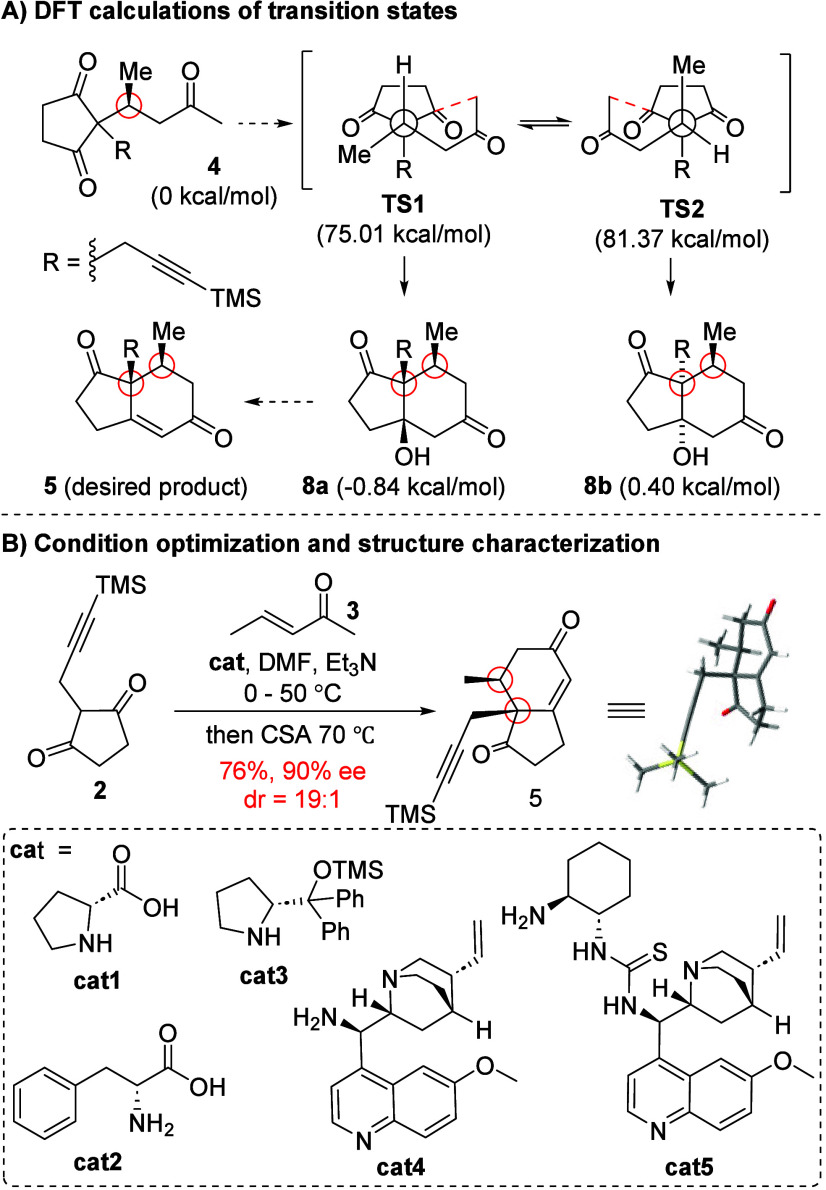
Studies of the Diastereo-/Enantioselective HPESW Reaction

Following the theoretical study, the diastereo- and enantioselectivity of the HPESW reaction were studied using various chiral amino acid^23–27^ and cinchona derivatives^28–30^ as the catalyst (Scheme 1B). Unfortunately, using **cat1**–**cat4** as the catalyst generally resulted in low diastereoselectivity, with **5** obtained only as the minor diastereomeric product, indicating a mismatch scenario of chirality between the catalyst and the stereochemistry of intermediate **4** during the intramolecular aldol cyclization step. Gratifyingly, **cat5** provided the desired cyclized product **5** as the major diastereomer. After a systematic investigation of the effects of catalyst loading, solvent, additives, and reaction temperature, the reaction condition was successfully optimized using 20 mol % of **cat5** with 40 mol % of Et_3_N in DMF from 0 to 50 °C, which afforded **5** in 76% yield, 90% ee, and a dr of 19:1. Preliminary equilibration studies showed that both **cat5** and Et_3_N at high reaction temperature are important for achieving the high diastereoselectivity of **5**, indicating the equilibration of the intramolecular aldol cyclization step. Detailed mechanistic studies are still ongoing. The structure of compound **5** was fully characterized by X-ray crystallography.

With **5** in hand, C9-methyl was introduced by methyl cuprate addition. Unfortunately, the C6-ketone became inactive toward a variety of nucleophilic reagents due to the steric hindrance of the C9-methyl. Because of this, the C2-ketone was selectively protected as 1,3-dithinolane with 1,2-ethanethiol and BF_3_·OEt_2_ in MeOH, yielding dithiolane **9** in 90% yield. Wittig olefination of **9** with (methoxymethyl)­triphenylphosphonium chloride and *t*-BuOK, followed by acid hydrolysis, provided aldehyde **10** in 70% yield as a single diastereomer. Subsequent Wittig olefination installed the dimethyl alkenyl side chain at C6, completing the synthesis of PK cyclization precursor **6**. According to the literature, the intramolecular PK reaction of geminal dimethyl alkene with terminal alkyne has not been reported. Inspired by Yang’s work on Rh-catalyzed PK reaction for establishing the quaternary carbon of the α-dimethyl cyclopentenone,^31^ cyclization precursor **6** was treated with Rh­(CO)_2_Cl_2_/CO in 1,2-dichloroethane at 60 °C. However, this condition resulted in only trace amounts of the desired product along with several unidentifiable side-products. Switching to PdCl_2_/LiCl with tetramethyl thiourea^31^ resulted in no reaction, and slow decomposition of **9** was observed at high reaction temperatures. These results indicated that the Rh and Pd catalysts may have interactions with the dithiane moiety of **6**, causing side reactions or decomposition of the substrate. After a systematic survey of different catalysts and conditions,^25,32–36^ we successfully optimized the PK reaction with Co­(CO)_8_/NMO in CH_2_Cl_2_ at 0 °C to rt, which afforded the ABCD tetracyclic ring system of **7** in 60% yield. The structures of **7** were unambiguously characterized by X-ray crystallography. Starting from HPESW ketone **5**, we successfully established the tetracyclic cyclopiane core in only four steps with 36% overall yield.

After establishing the cyclopiane skeleton, methyl cuprate addition of **7** followed by *in situ* reduction of the C13-ketone using lithium tri-*tert*-butoxyaluminum hydride (LTBA) provided alcohol **11** in 90% yield as a single diastereomer (Scheme 2). Deprotection of the 1,3-dithinolane and protection of the C13-hydroxyl as a TES ether resulted in enone **12**. Unfortunately, attempts to install the C9-methyl through various 1,4-addition conditions, including different cuprate and Ni-catalyzed nucleophilic additions,^37–42^ were unsuccessful. According to the preliminary conformational analysis (inset in Scheme 2), the convex face (top face) of **12** was highly shielded by the C4-methyl and the CD bicyclic ring system, preventing the attack of the nucleophile (Nu^–^) from the top face and the formation of the *cis*-fused AB ring system. We then switched our attention to the cyclopropane formation/ring-opening strategy.^31,43,44^ LTBA reduction of enone **12**, followed by the hydroxyl-directed Simmons–Smith reaction, afforded cyclopropane **13** in 82% yield over two steps with a dr of 9:1. Ley-Griffith oxidation of the C2-hydroxyl provided ketone **14**. The stereochemistry of C13-hydroxyl was determined by X-ray crystallography of compound **15**, which was prepared from ketone **14** via desilylation and acylation of the C13-hydroxyl. Cyclopropane ring-opening of ketone **14** under Birch reduction conditions successfully installed the C9-methyl, resulting in **16**. Treatment with NaBH_4_ in THF and *in situ* addition of DIAD/PPh_3_ converted the C2-ketone to the alkene of **17** as a 6:1 regioisomeric mixture, which is not separable by silica gel column chromatography. Finally, *m*-CPBA epoxidation of **17**, followed by epoxide ring-opening with LAH, *in situ* desilylation with TBAF, and IBX oxidation, installed the enone moiety in the A ring, affording leptosphin C (**1**) in 55% yield over three steps. The NMR data of synthetic leptosphin C (**1**) matched those reported in the literature (Tables S1 and S2). A preliminary cytotoxic activity study of synthetic leptosphin C (**1**) using cell counting kit (CCK)-8 assays showed moderate activities against human lung adenocarcinoma cells (A549, IC_50_ = 60.95 μM) and promyelocytic leukemia cells (HL-60, IC_50_ = 92.04 μM) and mouse macrophage cells (RAW264.7, 33.09 μM). Related biological studies are ongoing in our laboratory and will be reported in due course.

**Scheme 2:**
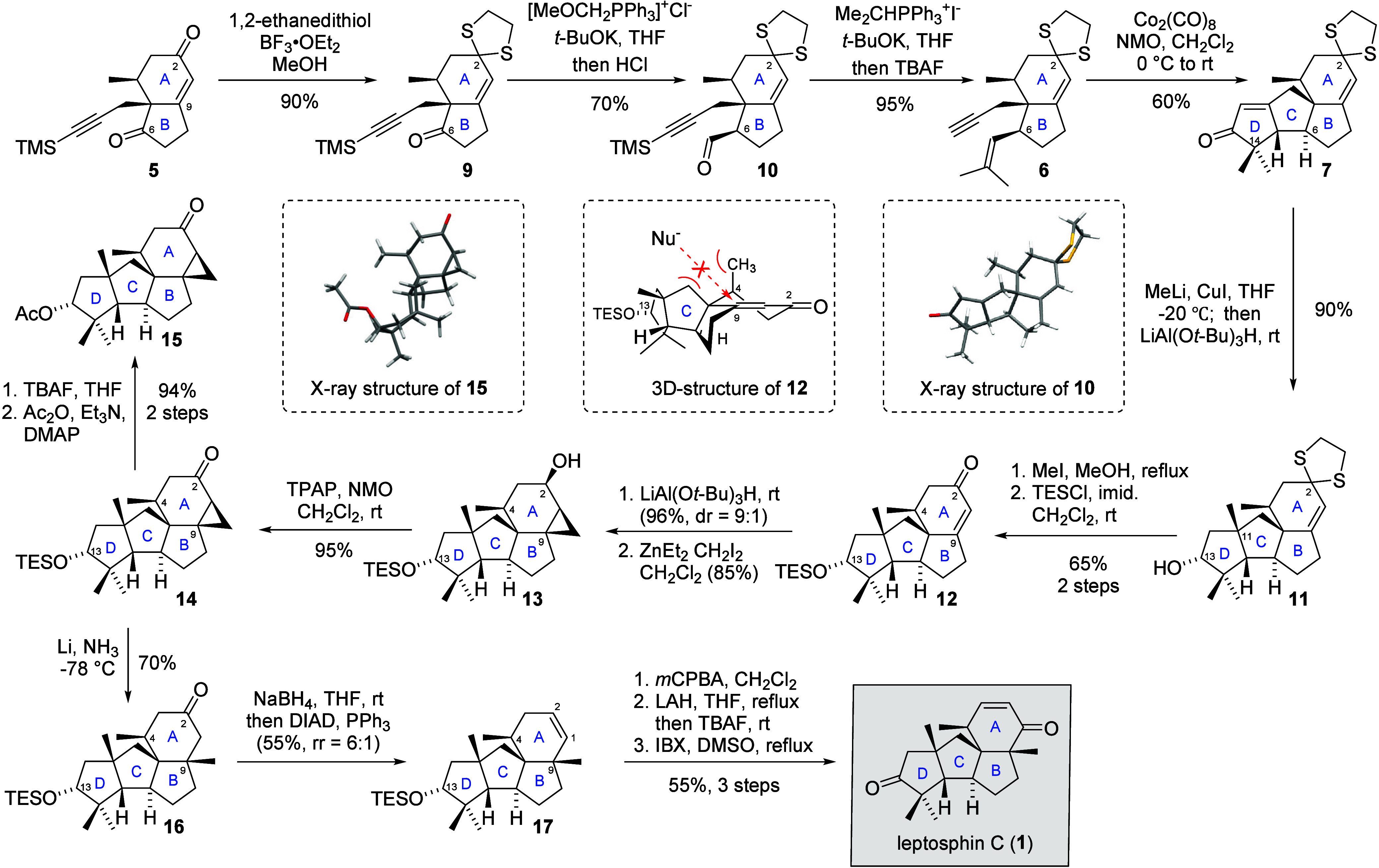
Asymmetric Total Synthesis of Leptosphin C (**1**)

In summary, we have achieved an asymmetric total synthesis of leptosphin C (**1**) through a diastereo-/enantioselective HPESW reaction. The chirality mismatch problem of the HPESW reaction was overcome by using cinchona-derived catalyst **cat5** (20 mol %) with Et_3_N (40 mol %) in DMF from 0 to 50 °C, which afforded the desired HPESW ketone **5** in 76% yield with a dr of 19:1 and 90% ee. The tetracyclic cyclopiane core was rapidly established from **5** in only four steps and the total synthesis of leptosphin C (**1**) was achieved in total in 16 steps from readily available substrates with 2.74% overall yield. Syntheses of other natural products bearing a 5,6-bicyclic ring system with continuous stereogenic centers such as waihoensene, dysiherbol A, and isoneoamphilectane utilizing this synthetic strategy are ongoing in our laboratory.

## Supplementary Material



## Data Availability

The data underlying this study are available in the published article and its Supporting Information.

## Notes

General information, experimental details, characterization data of new compounds, HPLC analysis of **5**, X-ray structures and crystallographic coordinates of **5**, **7**, and **15**, Gibbs energy profile for intramolecular aldol cyclization of **4**, *in vitro* assays of synthetic leptosphin C (**1**), and ^1^H and ^13^C NMR spectra of new compounds (PDF)
