# Supplementary material for: Asymmetric Total Synthesis of Leptosphin C
Source: Org Lett. 2025 Aug 26;27(35):9814–9. doi: 10.1021/acs.orglett.5c03236 (PMC12418496; doi:10.1021/acs.orglett.5c03236)
Supplement: Supplementary file 1 [file ol5c03236_si_001.pdf]

Supporting Information

Asymmetric Total Synthesis of Leptosphin C

Lantian Sun, [†,a] Chenshan Lian, [†,b] Jingyi Zhao,[a] Xiaole Chen,[a] Wei Han,*[,a] Zigang Li,*[,b] Chi-Sing Lee*[,a]

[a] Department of Chemistry, Hong Kong Baptist University, Waterloo Road, Kowloon Tong, Hong Kong SAR 999077, China

[b] State Key Laboratory of Chemical Oncogenomics, School of Chemical Biology and Biotechnology, Peking University Shenzhen Graduate School, Shenzhen University Town, Xili, Shenzhen 518055, China.

[†] These authors contributed equally to this work.

Table of Contents

I. General Information for synthesis...S3

II. Synthesis of new compounds...S3-S13

Table S1. Comparison of the ^{1}H NMR ($CDCl_3$) signals of natural and synthetic leptosphin C……………S12

Table S2. Comparison of the $^{13}C\{^{1}H\}$ NMR ($CDCl_3$) siganls of natural and synthetic leptosphin C……..S13

III. X-ray structures...S14-S17

Table S3. Summary of crystal data and refinement parameters for compounds **5**, **7** and **15...**……………..S17

IV. HPLC analysis……………………………………………………………………………S18-19

V. DFT calculations...S20-S26

Figure S1. Gibbs energy profile for intramolecular aldol cyclization of **4**……………………………………S20

Table S4. Calculated imaginary frequencies of all transition states species.……………………………....S21

Table S5. Calculated energy values of all species…………………………………………………….....S21

Table S6. Calculated energy values of all species at M06-2X/def2-TZVP………………………………...S21

Table S7– S11. Coordinates (Å) for the optimized structures of all species………………………….S22-S26

VI. General Information for *in vitro* assays ……………………….…………………..…………...S27

Figure S2. CCK8 assays of leptosphin C (**1**)………………………………………………………….S27

VII. NMR Spectra of new compounds S28-S41

VIII. References S42

I. General Information for synthesis

All air and water sensitive reactions were carried out under a nitrogen atmosphere with dry solvents under anhydrous conditions, unless otherwise noted. All the chemicals were purchased commercially and used without further purification. Anhydrous THF and was distilled from sodium-benzophenone, toluene was distilled from sodium, and dichloromethane was distilled from calcium hydride. Yields refer to chromatographically, unless otherwise stated. Reactions were monitored by thin-layer chromatography (TLC) carried out on 0.25 mm silica gel plates (60F-254) that were analyzed by staining with $KMnO_4$ (200 mL H_2O of 1.5 g $KMnO_4$, 10 g K_2CO_3 and 1.25 mL of 10% aq NaOH), fluorescence upon 254 nm irradiation or by staining with anisaldehyde (450 mL of 95% EtOH, 25 mL of conc. H_2SO_4, 15 mL of acetic acid, and 25 mL of anisaldehyde). Silica gel (60, particle size 0.040–0.063 mm) was used for flash chromatography. IR spectra were obtained using FT-IR Spectrometer. NMR spectra were recorded on either a 300 (^{1}H: 300 MHz, ^{13}C: 75 MHz), 400 (^{1}H: 400 MHz, ^{13}C: 100 MHz), or 500 (^{1}H: 500 MHz, ^{13}C: 125 MHz). The following abbreviations were used to explain the multiplicities: s = singlet, d = doublet, t = triplet, q = quartet, m = multiplet, b = broad. High resolution mass spectra were obtained from a MALDI-TOF mass spectrometer. Crystallographic data were obtained from a single crystal X-ray diffractometer (X'taLAB Synerger MicroMax-007HF). High performance liquid chromatography (HPLC) analysis of chiral compounds was performed on a HPLC instrcutment using a chiral column with *n*-hexane and isopropanol as the eluent. Optical rotations were measured on a polarimeter. Melting points were uncorrected and determined on a micro-melting point meter.

II. Synthesis of new compounds

Synthesis of 5

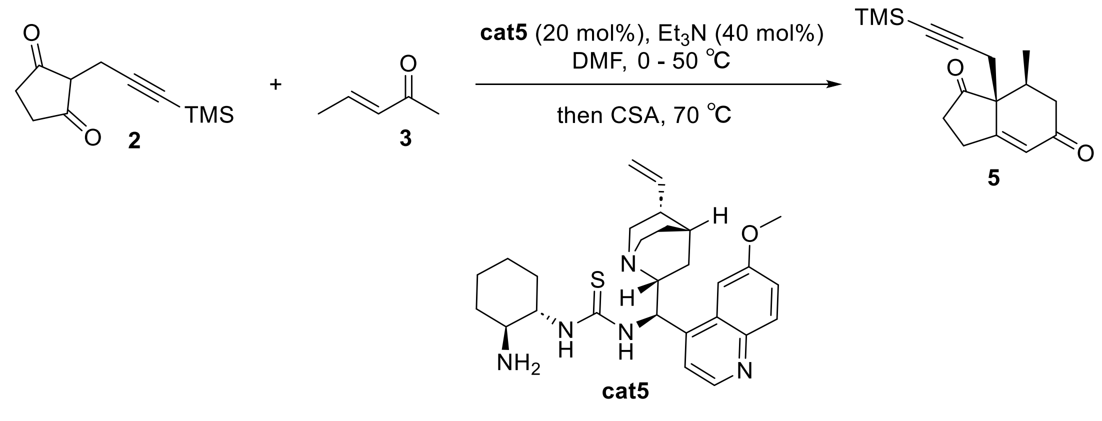

To a stirred solution of 1,3-diketone **2**[1] (6.3 g, 30.2 mmol), enone **3** (5.1 g, 60.4 mmol) and Et_3N (1.2 g, 12.1 mmol) in DMF (75 mL) was added **cat5**[2] (2.9 g, 6.0 mmol) at 0 °C. The resulting mixture was stirred at 0 °C for 1d, rt for 1 d and then 50 °C for 3 d. The mixture was then treated with CSA (10.5 g, 45.3 mmol) and the resulting

solution was stirred at 70 °C in an oil bath for 3 h. The reaction was quenched by addition of a saturated aqueous $NaHCO_3$ solution (225 mL) and the aqueous layer was extracted with a mixture of ethyl acetate and hexanes (1:1, 225 mL × 3). The combined organic extracts were washed with brine, dried over Na_2SO_4, filtered and concentrated. Silica gel flash column chromatography (ethyl acetate/hexanes = 1:6) of the residue gave a yellow amorphous solid (6.3 g, 23.0 mmol, 76%) as the product. **5**: $[\alpha]^{25}_{D}$ = -258.09° (c = 1.1, $CHCl_3$). ^{1}H NMR (400 MHz, $CDCl_3$): δ 6.12 (d, J = 2.6 Hz, 1H), 3.18-3.08 (m, 1H), 2.85-2.78 (m, 1H), 2.70-2.57 (m, 3H), 2.53-2.40 (m, 2H), 2.31-2.18 (m, 2H), 1.26 (d, J = 6.6 Hz, 3H), 0.10 (s, 9H). $^{13}C\{^1H\}$ NMR (100 MHz, $CDCl_3$): δ 215.7, 198.3, 168.6, 125.5, 101.4, 9.6, 53.9, 42.2, 37.5, 35.3, 28.8, 21.8, 15.6, -0.2. HRMS (ESI): Calcd for $C_{16}H_{23}O_2Si^+$ $[M+H]^+$: 275.1462; found: 275.1462.

Synthesis of 9

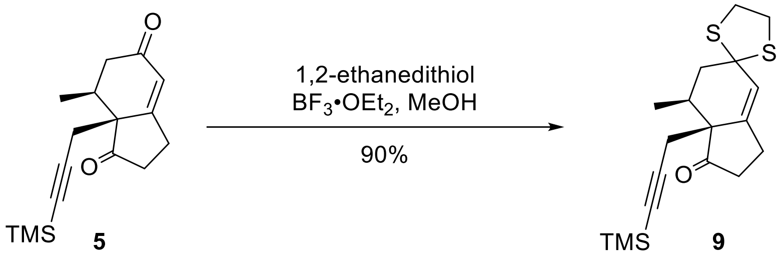

To a stirred solution of **5** (12.6 g, 45.9 mmol) and 1,2-ethanedithiol (4.8 g, 50.5 mmol) in MeOH (150 mL) was added BF_3•Et_2O (7.8 g, 55.1 mmol) at 0 °C. The mixture was then stirred at rt for 24 h. After removal of the solvent under vacuum, the residue was dissolve in a saturated aquoeus $NaHCO_3$ solution (50 mL), and the aqueous layer was extracted with ethyl acetate (50 mL × 3). The combined organic extracts were washed with brine, dried over Na_2SO_4, filtered and concentrated. Silica gel flash column chromatography (ethyl acetate/hexanes = 1:15) of the residue gave a yellow oil (14.5 g, 41.3 mmol, 90%) as the product. **9**: $[\alpha]^{25}_{D}$ = -211.9° (c = 1.0, $CHCl_3$). ^{1}H NMR (400 MHz, $CDCl_3$): δ 5.85 (s, 1H), 2.96-2.86 (m, 1H), 3.45-3.18 (m, 4H), 2.56-2.26 (m, 6H), 2.07-2.02 (m, 1H), 1.93-1.85 (m, 1H), 1.20 (d, J = 6.9 Hz, 3H), 0.13 (s, 9H). $^{13}C\{^1H\}$ NMR (100 MHz, $CDCl_3$): δ 217.4, 142.0, 127.6, 102.9, 88.5, 65.2, 52.5, 47.0, 40.8, 40.0, 37.9, 34.5, 27.8, 21.6, 15.8, 0.0. HRMS (ESI): Calcd for $C_{18}H_{27}OS_2Si^+$ $[M+H]^+$: 351.1267; found: 351.1268.

Synthesis of 10

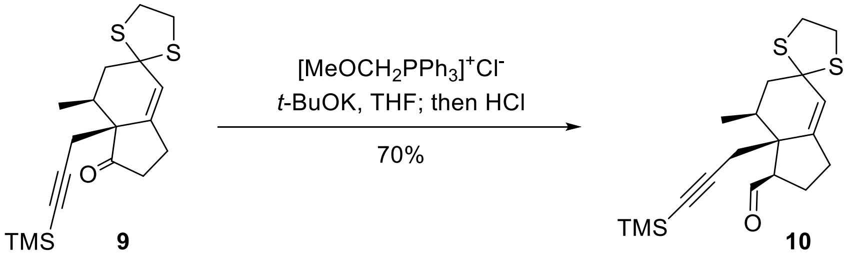

To a stirred solution of $[MeOCH_2PPh_3]^+Cl^-$ (28.3 g, 82.6 mmol) in THF (120 mL) was added *t*-BuOK (9.3 g, 82.6 mmol) at 0 °C, and the reaction mixture was stirred at rt for 2 h. A solution of **9** (14.5 g, 41.3 mmol) in THF (60 mL) was added slowly to the above stirred solution at 0 °C and the resulting mixture was stirred at rt for 4 h. The pH of the mixture was adjusted to 1 – 2 by addition of a 2 M aqueous of HCl solution (~100 mL). The resulting mixture was stirred at rt for 2 h and the reaction was quenched by addition of a saturated aqueous $NaHCO_3$ solution (60 mL). The aqueous layer was extracted with ethyl acetate (3 × 80 mL). The combined organic extracts were washed with brine (50 mL), dried over Na_2SO_4, and concentrated. Silica gel flash column chromatography (ethyl acetate/*n*-hexane 1:20) of the residue gave a yellow oil (10.5 g, 28.9 mmol, 70%) as the product. **10**: $[\alpha]^{25}_{D}$ = -143.3° (*c* = 1.2, $CHCl_3$). ^1H NMR (400 MHz, $CDCl_3$): δ 9.97 (d, *J* = 3.0 Hz, 1H), 5.63 (s, 1H), 3.45-3.19 (m, 4H), 2.79-2.70 (m, 1H), 2.54-2.33 (m, 5H), 2.28-2.18 (m, 1H), 2.08-2.04 (m, 1H), 1.95-1.84 (m, 2H), 1.09 (d, *J* = 7.0 Hz, 3H), 0.14 (s, 9H). $^{13}C\{^1H\}$ NMR (100 MHz, $CDCl_3$): δ 203.8, 146.8, 125.1, 104.6, 88.6, 65.8, 62.8, 51.6, 47.1, 41.3, 40.7, 39.9, 29.4, 22.5, 20.3, 17.5. HRMS (ESI): Calcd for $C_{19}H_{29}OS_2Si^+$ $[M+H]^+$: 365.1424; found: 365.1425.

Synthesis of 6

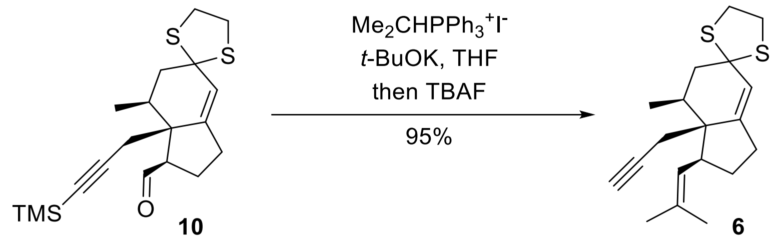

To a stirred solution of $Me_2CHPPh_3^+Cl^-$ (25.0 g, 57.8 mmol) in THF (100 mL) was added *t*-BuOK (6.5 g, 57.8 mmol) at 0 °C. The mixture was stirred at rt for 2 h. A solution of **10** (10.5 g, 28.9 mmol) in THF (50 mL) was added slowly into the above stirred solution at 0 °C. The resulting mixture was stirred at rt for 1 h. TLC analysis showed partial hydrolysis of the TMS group. The mixture was then treated with TBAF (31.8 mL, 31.8 mmol, a 1 M solution in THF). After stirring at rt for 1 h, the reaction was quenched by addition of water (150 mL) and washed with brine (100 mL), dried over Na_2SO_4, and concentrated. Silica gel column chromatography (ethyl acetate/*n*-hexane 1:50) of the residue gave a yellow oil (8.7 g, 27.5 mmol, 95%) as the product. **6**: $[\alpha]^{25}_{D}$ = +83.4° (*c* = 9.2, $CHCl_3$). ^1H NMR (400 MHz, $CDCl_3$): δ 5.6 (s, 1H), 5.08 (d, *J* = 10.1 Hz, 1H), 3.38-3.17 (m, 4H), 2.75-2.68 (m, 1H), 2.56-2.47 (m, 1H), 2.44-2.37 (m, 1H), 2.32-2.15 (m, 3H), 1.96-1.91 (m, 2H), 1.79-1.70 (m, 2H), 1.67 (s, 3H), 1.60 (s, 3H), 1.54-1.43 (m, 1H), 0.94 (d, *J* = 6.9 Hz, 3H). $^{13}C\{^1H\}$ NMR (100 MHz, $CDCl_3$): δ 147.5, 132.9, 125.5, 124.6, 83.4, 70.7, 66.2, 50.4, 49.e, 47.8, 40.7, 40.4, 39.8, 28.6, 28.5, 26.2, 18.3, 17.2, 15.9. HRMS (ESI): Calcd for $C_{19}H_{27}S_2^+$ $[M+H]^+$: 319.1549; found: 319.1549.

Synthesis of 7

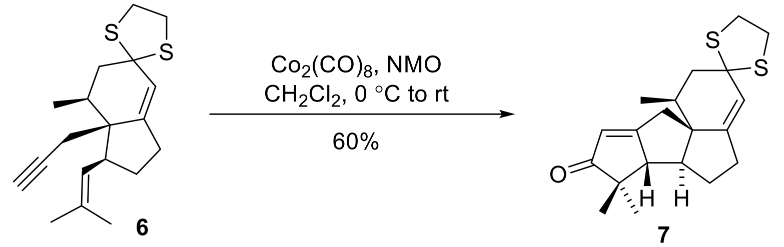

To a stirred solution of **6** (2.0 g, 6.3 mmol) in CH_2Cl_2 (210 mL) was added $Co_2(CO)_8$ (2.2 g, 6.9 mmol) at 0 °C, and the reaction mixture was then stirred at rt for 2 h. To this solution was added NMO (7.4 g, 63 mmol) at 0 °C. The resulting mixture was allowed to generally warmed to rt, and stirred for 24 h. After removal of the solvent under cacuum, silica gel flash column chromatography (ethyl acetate/hexanes = 1:6) of the residue gave a white amorphous solid (1.3 g, 3.8 mmol, 60%) as the product. **7**: $[\alpha]^{25}_D$ = -52.4° (c = 1.2, $CHCl_3$). ^1H NMR (400 MHz, $CDCl_3$): δ 5.71 (s, 1H), 5.56 (s, 1H), 3.47-3.19 (m, 4H), 2.83 (d, J = 16.6 Hz, 1H), 2.53-2.39 (m, 2H), 2.27-2.18 (m, 4H), 2.13-2.06 (m, 1H), 1.74-1.69 (m, 1H), 1.63-1.52 (m, 2H), 1.16 (s, 3H), 1.05 (s, 3H), 0.76 (d, J = 6.9 Hz, 3H). $^{13}C\{^1H\}$ NMR (100 MHz, $CDCl_3$): δ 215.6, 186.0, 149.0, 122.1, 120.6, 65.8, 64.3, 56.2, 48.4, 48.2, 45.0, 40.8, 39.9, 39.39, 35.9, 31.5, 31.3, 25.0, 22.3, 16.6. HRMS (ESI): Calcd for $C_{20}H_{27}OS_2^+$ $[M+H]^+$: 347.1498; found: 347.1498.

Synthesis of 11

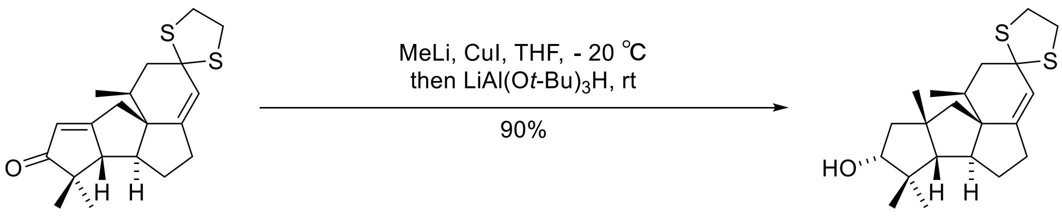

To a stirred solution of CuI (628.5 mg, 3.3 mmol) in THF (10 mL) was added MeLi (4.1 mL, 6.6 mmol, 1.6 M in diethyl ether) at -20 °C, and the reaction mixture was stirred at -20 °C for 0.5 h. To this solution was added enone **7** (770.0 g, 2.2 mmol) in THF (5 mL) at -20 °C, and the reaction mixture was then stirred at -20 °C for 2 h. To this stirred solution was added t-BuOH (81.5 mg, 1.1 mmol) at -20 °C and stirred at -20 °C for 10 min. After addition of LiAl(Ot-Bu)$_3$H (2.8 g, 11.0 mmol) at 0 °C, the resutling mixture was stirred at rt for 15 h. The reaction was then quenched by addition of a saturated aqueous sodium tartrate solution (15 mL) and the aqueous layer was extracted with ethyl acetate (15 mL × 3). The combined organic extracts were washed with brine (25 mL), dried over Na_2SO_4, and concentrated. Silica gel column chromatography (ethyl acetate/n-hexane 1:5) of the residue gave a colourless oil (721.9 mg, 2.0 mmol, 90%) as the product. **11**: $[\alpha]^{25}_D$ = +16.0° (c = 0.5, $CHCl_3$). ^1H NMR (400 MHz, $CDCl_3$): δ 5.46 (s, 1H), 3.75 (dd, J = 9.0, 6.0 Hz, 1H), 3.45-.21 (m, 4H), 2.54 (tdd, J = 13.5, 6.5 Hz, 2.3 Hz, 1H), 2.48 (t, J = 6.1 Hz, 1H), 2.22-2.14 (m, 2H), 2.11-2.05 (m, 1H), 2.00 (d, J = 13.7 Hz, 1H), 1.80 (dd,

J = 12.5 Hz, 5.9 Hz, 1H), 1.64-1.45 (m, 5H), 1.41-1.38 (m, 1H), 1.12 (s, 3H), 0.99 (s, 3H), 0.98 (d, J = 6.8 Hz, 3H), 0.91 (s, 3H). $^{13}C\{^1H\}$ NMR (100 MHz, $CDCl_3$): δ 155.0, 121.3, 82.0, 71.1, 65.9, 58.1, 50.5, 49.7, 48.9, 47.7, 45.8, 45.4, 40.9, 39.7, 38.8, 33.6, 33.2, 32.2, 29.8, 18.9, 17.1. HRMS (ESI): Calcd for $C_{21}H_{33}OS_2^+$ $[M+H]^+$: 365.1967; found: 365.1968.

Synthesis of 12

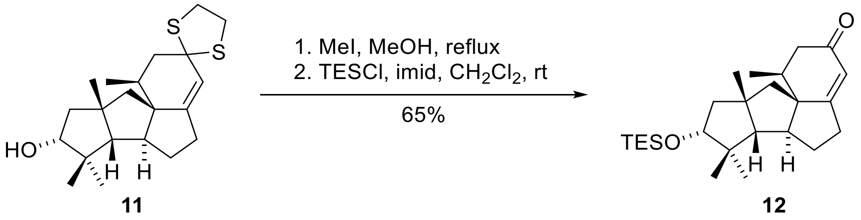

To a stirred solution of **11** (721.9 mg, 2.0 mmol) in MeOH (6 mL) was added MeI (2.8 g, 20.0 mmol) at rt. The mixture was heated under reflux in an oil bath for 4 h. After removal of the solvent under vacuum, the residue was dissolved in CH_2Cl_2 (5 mL) and treated with imidazole (272.0 mg, 4.0 mmol and TESCl (602.9 mg, 4.0 mmol) at rt, The resulting mixture was stirred at rt for 5 h. The reaction was quenched by addition of water (10 mL) and the aqueous layer was extracted with ethyl acetate (3 × 10 mL). The combined organic extracts were washed with brine (20 mL), dried over Na_2SO_4, and concentrated. Silica gel column chromatography (ethyl acetate/*n*-hexane 1:12) of the residue gave a colourless oil (523.5 mg, 1.3 mmol, 65%) as the product. **12**: $[\alpha]^{25}_D$ = -70.0° (c = 1.2, $CHCl_3$). ^1H NMR (400 MHz, $CDCl_3$): δ 5.79 (d, J = 2.5 Hz, 1H), 3.69 (dd, J = 8.5, 6.0 Hz, 1H), 2.91-2.81 (m, 1H), 2.63 (d, J = 5.7 Hz, 1H), 2.43 (dd, J = 15.7, 5.8 Hz, 1H), 2.28- 2.22 (m, 2H), 2.11-2.03 (m, 1H), 2.01-1.93 (m, 1H), 1.76-1.58 (m, 5H), 1.47 (d, J = 5.5 Hz, 1H), 1.07 (s, 3H), 1.06 (d, J = 6.6 Hz, 3H), 0.97-0.92 (m, 15H), 0.56 (q, J = 7.9 Hz, 6H). $^{13}C\{^1H\}$ NMR (100 MHz, $CDCl_3$): δ 200.6, 182.6, 120.9, 81.8, 70.0, 61.1, 49.9, 49.8, 46.7, 46.4, 46.0, 44.2, 39.5, 34.4, 32.3, 32.2, 29.5, 18.9, 16.7, 7.0, 5.1. HRMS (ESI): Calcd for $C_{25}H_{43}O_2Si^+$ $[M+H]^+$: 403.3027; found: 403.3028.

Synthesis of 18

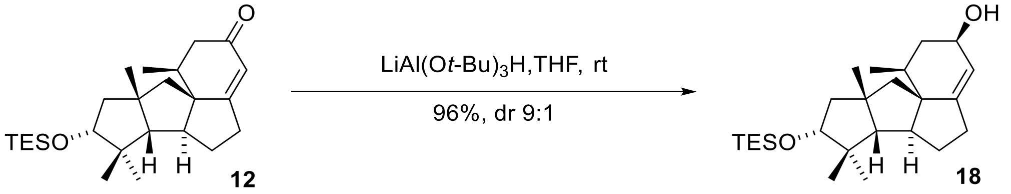

To a stirred solution of **12** (310.0 mg, 0.8 mmol) in THF (9 mL) was added LiAl(O*t*-Bu)$_3$H (610.2 mg, 2.4 mmol) at rt and the mixture was stirred at rt for 12 h. The reaction was quenched by addition of a saturated aqueous sodium tartrate solution (10 mL) and the aqueous layer was extracted with ethyl acetate (3 × 10 mL). The combined organic extracts were washed with brine (15 mL), dried over Na_2SO_4, and concentrated. Silica gel

column chromatography (ethyl acetate/*n*-hexane 1:5) of the residue gave a colourless oil (311.6 mg, 0.77 mmol, 96%) as the product. **18**: $[\alpha]^{25}_D$ = -46.9° (*c* = 1.3, $CHCl_3$). ^1H NMR (400 MHz, $CDCl_3$): δ 5.41 (d, *J* = 4.0 Hz, 1H), 4.30 (q, *J* = 6.9 Hz, 1H), 3.69 (dd, *J* = 9.3, 6.2 Hz, 1H), 2.62-2.55 (m, 1H), 2.48 (t, *J* = 5.8 Hz, 1H), 2.16 (dd, *J* = 13.9, 5.3 Hz, 1H), 2.07 (d, *J* = 13.6 Hz, 1H), 1.97-1.92 (m, 1H), 1.70-1.67 (m, 2H), 1.64-1.60 (m, 1H), 1.51-1.46 (m, 2H), 1.45-1.39 (m, 3H), 1.33-1.28 (m, 1H), 1.10 (s, 3H), 0.98-0.93 (m, 15H), 0.88 (s, 3H), 0.57 (q, *J* = 8.5 Hz, 6H). $^{13}C\{^1H\}$ NMR (100 MHz, $CDCl_3$): δ 159.6, 119.14, 81.7, 70.3, 67.8, 59.1, 51.1, 49.7, 47.5, 46.0, 45.4, 40.3, 39.7, 33.7, 32.8, 32.4, 29.7, 19.0, 17.7, 7.1, 5.1. HRMS (ESI): Calcd for $C_{25}H_{44}NaO_2Si^+$ $[M+Na]^+$: 427.3003; found: 427.3009.

Synthesis of 13

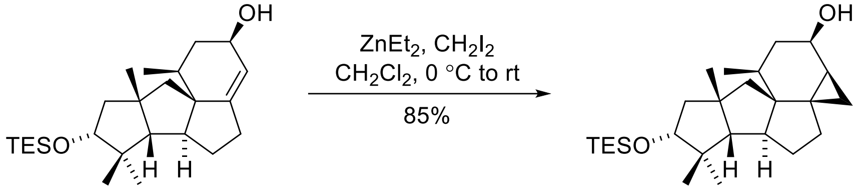

18 **13**

To a stirred solution of **18** (311.6 mg, 0.77 mmol) and CH_2I_2 (1.0 g, 3.8 mmol) in CH_2Cl_2 (3 mL) was added $ZnEt_2$ (3.8 mL, 3.8 mmol, a 1 M solution in toluene) at 0 °C. The mixture was stirred at the rt for 8 h. The reaction was then quenched by addition of a saturated aqueous NH_4Cl solution (10 mL) and the aqueous layer was extracted with ethyl acetate (3 × 10 mL). The combined organic extracts were washed with brine (10 mL), dried over Na_2SO_4, and concentrated under vacuum. Silica gel column chromatography (ethyl acetate/*n*-hexane 1:5) of the residue gave a colourless oil (272.2 mg, 0.65 mmol, 85%) as the product. **13**: $[\alpha]^{25}_D$ = -19.0° (*c* = 2.7, $CHCl_3$). ^1H NMR (400 MHz, $CDCl_3$): δ 4.19-4.14 (m, 1H), 3.66 (dd, *J* = 8.1, 5.9 Hz, 1H), 2.48 (t, *J* = 5.9 Hz, 1H), 2.13 (td, *J* = 13.0, 6.0 Hz, 1H), 1.75-1.68 (m, 3H), 1.61-1.57 (m, 1H), 1.41-1.28 (m, 5H), 1.27-1.20 (m, 2H), 1.18 (s, 3H), 1.16-1.13 (m, 1H), 0.96-0.86 (m, 18H), 0.60-0.53 (m, 7H), 0.53-0.48 (m, 1H). $^{13}C\{^1H\}$ NMR (100 MHz, $CDCl_3$): δ 82.1, 68.8, 67.8, 57.5, 50.6, 50.2, 46.2, 46.1, 45.1, 38.2, 4.16, 37.0, 35.8, 31.4, 31.1, 30.0, 29.5, 18.7, 17.9, 12.1, 7.04, 5.0. HRMS (ESI): Calcd for $C_{26}H_{46}NaO_2Si^+$ $[M+Na]^+$: 441.3159; found: 441.3162.

Synthesis of 14

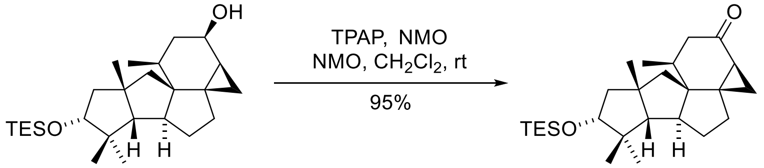

13 **14**

To a stirred solution of **13** (276.3 mg, 0.66 mmol) in CH_2Cl_2 (3 mL) was added TPAP (23 mg, 0.066 mmol) and NMO (154.6 mg, 1.3 mmol) at 0 °C, and the reaction mixture was then stirred at rt for 8 h. After removal of

solvent under vacuum, silica gel column chromatography (ethyl acetate/*n*-hexane 1:10) of the residue gave a colourless oil (261.3 mg, 0.63 mmol, 95%) as the product. **14**: $[\alpha]^{25}_{D}$ = -20.0° (*c* = 1.0, $CHCl_3$). ^1H NMR (400 MHz, $CDCl_3$): δ 3.69 (dd, *J* = 7.1, 5.7 Hz, 1H), 2.60 (t, *J* = 6.0 Hz, 1H), 2.21 (td, *J* = 13.0, 6.0 Hz, 1H), 1.93 (d, *J* = 13.3 Hz, 1H), 1.87-1.73 (m, 5H), 1.67-1.61 (m, 2H), 1.49-1.37 (m, 3H), 1.33-1.24 (m, 3H), 1.22 (s, 3H), 1.17-1.14 (m, 1H), 0.96-0.92 (m, 18H), 0.59-0.54 (m, 6H). $^{13}C\{^1H\}$ NMR (100 MHz, $CDCl_3$): δ 211.0, 82.4, 67.7, 58.1, 51.1, 50.2, 47.0, 46.4, 44.7, 43.1, 42.7, 41.7, 36.7, 36.4, 31.6, 30.8, 29.8, 21.6, 19.0, 18.0, 7.1, 5.0. HRMS (ESI): Calcd for $C_{26}H_{45}O_2Si^+$ $[M+H]^+$: 417.3183; found: 417.3188.

Synthesis of 15

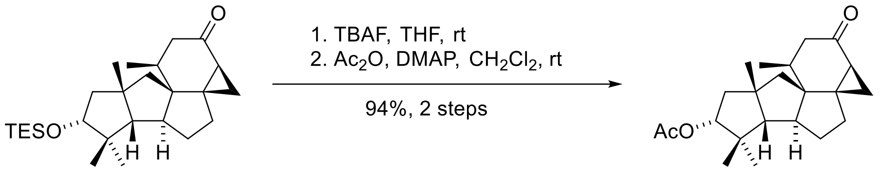

14 **15**

To a stirred solution of **14** (20.8 mg, 0.05 mmol) in THF (1 mL) was added TBAF (55 μL, 0.055 mmol, 1.0 M in THF) at 0 °C. The resulting mixture was stirred at 0 °C for 1 h and the reaction was quenched by addition of water (2 mL). The aqueous layer was extracted with a ethyl acetate/hexanes mixture (1:1, 2 mL × 3). The combined organic extracts were washed with brine, dried over Na_2SO_4, filtered and concentrated. To a stirred solution of the above crude product in CH_2Cl_2 (1 mL) was added Ac_2O (6.1 mg, 0.06 mmol), TEA (10.1 mg, 0.1 mmol) and DMAP (0.6 mg, 0.005 mmol) at rt. The resulting mixture was stirred at rt for 1 h and the reaction was quenched by addition of a saturated aqueous $NaHCO_3$ solution (2 mL). The aqueous layer was extracted with CH_2Cl_2 (2 mL × 3). The combined organic extracts were washed with brine, dried over Na_2SO_4, filtered and concentrated. Silica gel flash column chromatography (ethyl acetate/hexanes = 1:10) of the residue gave a white solid (16.2 mg, 0.047 mmol, 89%) as the product for three steps. **15**: $[\alpha]^{20}_{D}$ = -5.7° (*c* = 1.8, $CHCl_3$). ^1H NMR (400 MHz, $CDCl_3$): δ 4.89 (dd, *J* = 5.6, 4.0 Hz, 1H), 2.64 (t, *J* = 6.2 Hz, 1H), 2.23 (td, *J* = 13.1, 6.1 Hz, 1H), 2.02 (s, 3H), 1.99-1.98 (m, 1H), 1.95-1.94 (m, 1H), 1.88-1.79 (m, 3H), 1.77-1.76 (m, 1H), 1.73-1.71 (m, 1H), 1.68 (q, *J* = 5.1 Hz, 1H), 1.53 (d, *J* = 2.3 Hz, 1H), 1.51 (d, *J* = 9.1 Hz, 1H), 1.44 (dd, *J* = 12.4, 6.0 Hz, 1H), 1.35 (q, *J* = 6.5 Hz, 1H), 1.30 (s, 3H), 1.28 (d, *J* = 5.4 Hz, 1H), 1.17 (t, *J* = 5.2 Hz, 1H), 1.02 (s, 3H), 1.01-1.00 (m, 6H). $^{13}C\{^1H\}$ NMR (100 MHz, $CDCl_3$): δ 210.6, 170.7, 84.8, 68.1, 57.6, 51.8, 48.8, 47.0, 45.8, 44.63, 42.8, 42.6, 41.3, 36.3, 31.5, 30.6, 21.5, 19.7, 18.0. HRMS (ESI): Calcd for $C_{22}H_{33}O_3^+$ $[M+H]^+$: 345.2424; found: 345.2425.

Synthesis of 16

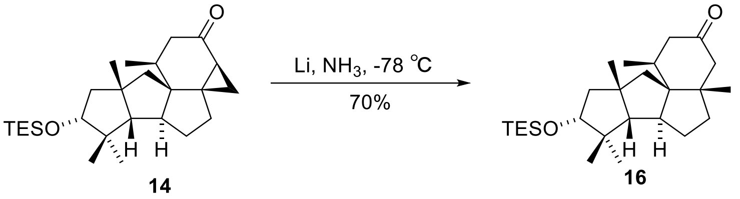

To a stirred dark blue solution of Li (44.1 mg, 6.3 mmol) in liquid NH_3 at -78 °C was slowly added a solution of **14** (261.3 mg, 0.63 mmol) in THF (2 mL). The resulting mixture was then stirred at -78 °C for 9 h. The reaction was quenched by slow addition of a saturated aqueous NH_4Cl solution (1.0 mL) until no blue color was observed. The mixture was allowed to warm to 25 °C slowly and stirred until the excess NH_3 was evaporated. The aqueous layer was extracted with ethyl acetate (3 × 3 mL). The combined organic extracts were washed with brine (3 mL), dried over Na_2SO_4, and concentrated. Silica gel flash column chromatography (ethyl acetate/*n*-hexane 1:10) of the residue gave a colourless oil (167.5 mg, 0.4 mmol, 70%) as the product. **16**: $[\alpha]^{25}_D$ = -26.7° (*c* = 1.2, $CHCl_3$). ^{1}H NMR (400 MHz, $CDCl_3$): δ 3.79 (t, *J* = 6.6 Hz, 1H), 2.66 (td, *J* = 8.5, 2.9 Hz, 1H), 2.38 (dd, *J* = 14.9, 1.5 Hz, 1H), 2.20-2.11 (m, 2H), 2.08-2.02 (m, 1H), 1.96-1.92 (m, 1H), 1.89-1.66 (m, 6H), 1.65-1.60 (m, 1H), 1.51 (d, *J* = 8.7 Hz, 1H), 1.42-1.35 (m, 1H), 1.20 (s, 3H), 1.07-1.06 (m, 6H), 0.99-0.90 (m, 15H), 0.59-0.52 (m, 6H). $^{13}C\{^1H\}$ NMR (100 MHz, $CDCl_3$): δ 213.3, 82.5, 71.6, 60.9, 53.7, 52.1, 50.8, 49.5, 48.3, 45.9, 45.0, 42.6, 41.2, 37.5, 31.96, 30.7, 30.5, 28.4, 21.4, 18.2, 7.0, 5.0. HRMS (ESI): Calcd for $C_{26}H_{47}O_2Si^+$ $[M+H]^+$: 419.3340; found: 419.3340.

Synthesis of 17

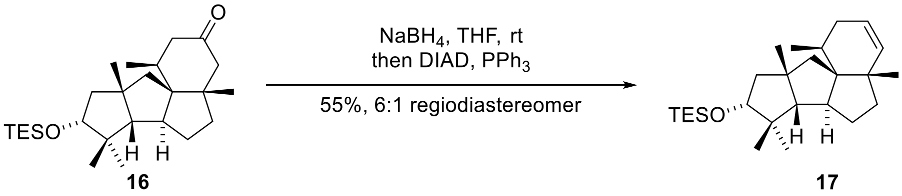

To a stirred solution of **16** (83.7 mg, 0.2 mmol) in THF (2 mL) was slowly added $NaBH_4$ (7.6 mg, 0.2 mmol) at rt. After stirring at rt for 1 h, DIAD (161.8 mg, 0.8 mmol) and PPh_3 (209.6 mg, 0.8 mmol) was added at 0 °C, and the resulting mixture was stirred at rt for 12 h. The reaction was then quenched by addition of a saturated aqueous NH_4Cl solution (1.0 mL) and the aqueous layer was extracted with ethyl acetate (3 × 3 mL). The combined organic extracts were washed with brine (3 mL), dried over Na_2SO_4, and concentrated. Silica gel flash column chromatography (ethyl acetate/*n*-hexane 1:30) of the residue gave a colourless oil (44.3 mg, 0.11 mmol, 55%) as the product (6:1 regiodiastereomer). **17**: $[\alpha]^{25}_D$ = -49.0° (*c* = 1.1, $CHCl_3$). ^{1}H NMR (400 MHz, $CDCl_3$): δ 5.55-5.51 (m, 1H), 5.26 (ddd, *J* = 9.9, 2.7, 1.2 Hz, 1H), 3.73 (dd, *J* = 7.8, 6.0 Hz, 1H), 2.51-2.48 (m, 1H), 2.26-2.20 (m, 1H), 1.96-1.93 (m, 1H), 1.87 (d, *J* = 14.6 Hz, 1H), 1.80-1.75 (m, 2H), 1.72-1.63 (m, 5H), 1.37-1.33 (m, 2H), 1.18 (s, 3H), 1.08 (s, 3H), 0.98-0.94 (m, 12H), 0.92 (d, *J* = 7.0 Hz, 3H), 0.89 (s, 3H), 0.60-0.54 (m, 6H).

$^{13}C\{^1H\}$ NMR (100 MHz, $CDCl_3$): δ 138.3, 123.1, 82.4, 73.1, 60.1, 53.1, 50.9, 48.0, 45.7, 45.6, 40.8, 36.8, 33.3, 31.7, 30.6, 30.6, 29.9, 25.0, 20.4, 17.5, 7.1, 5.1. HRMS (ESI): Calcd for $C_{26}H_{47}OSi^+$ $[M+H]^+$: 403.3391; found: 403.3384.

Synthesis of Leptosphin C (1)

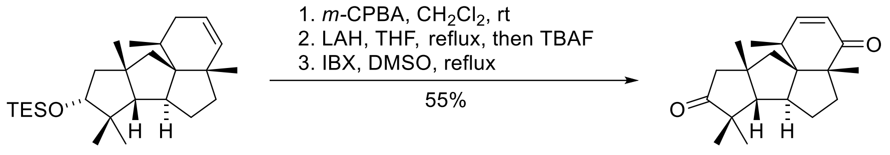

17 leptosphin C (**1**)

To a stirred solution of **17** (10.1 mg, 0.025 mmol) in CH_2Cl_2 (1 mL) was added *m*-CPBA (10.1 mg, 0.05 mmol, 85%) slowly at rt, and the mixture was at rt for 0.5 h. The reaction was quenched by addition of a saturated aqueous $NaHSO_3$ solution (0.5 mL) and a saturated aqueous $NaHCO_3$ solution (0.5 mL). The aqueous layer was extracted with ethyl acetate (3 × 1 mL). The combined organic extracts were washed with brine (1 mL), dried over Na_2SO_4, and concentrated. To a stirred solution of the above residue in THF (1 mL) was added $LiAlH_4$ (1.9 mg, 0.05 mmol) at rt, and the resulting mixture was heated under reflux for 6 h. After cooling to rt, TBAF (50 µL, 0.05 mmol, 1M in THF) was added and the mixture was stirred at rt for 0.5 h. The reaction was quenched by addition of water (1 mL) and the aqueous layer was extracted with ethyl acetate (3 × 1 mL). The combined organic extracts were washed with brine (1 mL), dried over Na_2SO_4, and concentrated. To a stirred solution of the above residue in DMSO (1 mL) was added IBX (35.0 mg, 0.125 mmol) at rt, and the mixture was then stirred at 80 °C in an oil bath for 12 h. After cooling to rt, the reaction was quenched by addition of a saturated aqueous $NaHCO_3$ solution (1 mL), and the aqueous layer was extracted with ethyl acetate (3 × 1 mL). The combined organic extracts were washed with brine (1 mL), dried over Na_2SO_4, and concentrated under vacuum. Silica gel flash column chromatography (ethyl acetate/*n*-hexane 1:10) of the residue gave a white amorphous solid (4.1 mg, 0.014 mmol, 55%) as the product. Leptosphin C (**1**): $[\alpha]^{25}_D$ = -66.6° (*c* = 0.12, $CHCl_3$). ^1H NMR (400 MHz, $CDCl_3$) δ 6.83 (dd, *J* = 10.1, 5.8 Hz, 1H), 5.96 (d, *J* = 10.1 Hz, 1H), 2.51 (d, *J* = 19.5 Hz, 1H), 2.35 (d, *J* = 19.4 Hz, 1H), 2.32-2.22 (m, 3H), 2.12 (d, *J* = 14.5 Hz, 1H), 1.90 (d, *J* = 4.6 Hz, 1H), 1.77 (m, 1H), 1.75 (m, 1H), 1.73 (m, 1H), 1.43 (s, 3H), 1.23 (m, 1H), 1.18 (d, *J* = 7.4 Hz, 3H), 1.17 (s, 6H), 1.01 (s, 3H). $^{13}C\{^1H\}$ NMR (100 MHz, $CDCl_3$): δ 223.7, 204.7, 153.2, 127.3, 70.0, 59.2, 57.2, 54.9, 50.9, 50.4, 46.6, 44.5, 37.7, 37.1, 33.5, 33.1, 29.5, 22.1, 21.6, 18.3. HRMS (ESI): Calcd for $C_{20}H_{29}O_2^+$ $[M+H]^+$: 301.2162; found: 301.2164.

Table S1. Comparison of ^{1}H NMR signals of natural and synthetic leptosphin C (**1**)

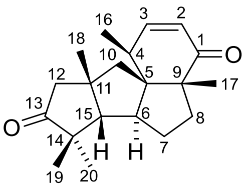

leptosphin C (**1**)

No.	Natural leptosphin C δ ^{1}H [ppm, mult, J (Hz)] 600 MHz	Synthetic leptosphin C δ ^{1}H [ppm, mult, J (Hz)] 400 MHz	Δδ[a] ppm
2	5.94 (d, 10.0, 1H)	5.96 (d, 10.1, 1H)	0.02
3	6.82 (dd, 5.8, 10.0, 1H)	6.83 (dd, 5.8, 10.1, 1H)	0.01
4	2.24 (m, 1H)	2.26 (m, 1H)	0.02
6	2.26 (m, 1H)	2.28 (m, 1H)	0.02
7α	1.22 (m, 1H)	1.23 (m, 1H)	0.01
7β	1.76 (m, 1H)	1.77 (m, 1H)	0.01
8α	2.22 (m, 1H)	2.24 (m, 1H)	0.02
8β	1.73 (m, 1H)	1.74 (m, 1H)	0.01
10α	1.70 (m, 1H)	1.72 (m, 1H)	0.01
10β	2.10 (d, 14.6, 1H)	2.12 (d, 14.5, 1H)	0.02
12α	2.34 (d, 19.5, 1H)	2.35 (d, 19.5, 1H)	0.01
12β	2.48 (d, 19.5, 1H)	2.51 (d, 19.5, 1H)	0.03
15	1.88 (d, 4.4, 1H)	1.90 (d, 4.6, 1H)	0.02
16	1.17 (d, 7.3, 3H)	1.18 (d, 7.4, 3H)	0.01
17	1.15 (s, 3H)	1.17 (s, 3H)	0.02
18	1.41 (s, 3H)	1.43 (s, 3H)	0.02
19	0.99 (s, 3H)	1.01 (s, 3H)	0.02
20	1.16 (s, 3H)	1.17 (s, 3H)	0.01

[a] Δδ (ppm) = Synthetic – Natural.

Table S2. Comparison of $^{13}C\{^{1}H\}$ NMR siganls of natural and synthetic leptosphin C (**1**)

No.	Natural leptosphin C<br>δ $^{13}C\{^{1}H\}$ [ppm, mult, J (Hz)]<br>150 MHz	Synthetic leptosphin C<br>δ $^{13}C\{^{1}H\}$ [ppm, mult, J (Hz)]<br>100 MHz	$\Delta\delta^{a}$ ppm
1	204.55	223.69	0.1
2	126.96	127.29	0.33
3	153.12	153.20	0.08
4	36.78	37.07	0.29
5	58.82	59.18	0.36
6	54.53	54.89	0.36
7	33.26	33.54	0.28
8	37.39	37.71	0.32
9	56.88	57.18	0.3
10	46.28	46.59	0.31
11	44.25	44.53	0.28
12	50.13	50.42	0.29
13	223.71	223.69	-0.02
14	50.65	50.88	0.23
15	69.65	70.03	0.38
16	18.07	18.31	0.24
17	21.41	21.63	0.22
18	32.87	33.08	0.21
19	21.90	22.14	0.24
20	29.27	29.51	0.24

[a] Δδ (ppm) = Synthetic – Natural.

III. X-ray structures

General procedures: 10 mg of a sample was weighted and transferred into a 2 mL sample vial. Then, 1 mL of *n*-hexane was added, and the mixture was slowly warmed to 50 °C. Ethyl acetate was gradually added dropwise to the solution until complete dissolution of the sample was achieved. After allowing the solution to cool to room temperature, the vial was capped and left undisturbed to enable slow crystallization of the product.

5 (CCDC 2414250)

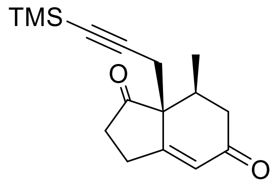

5

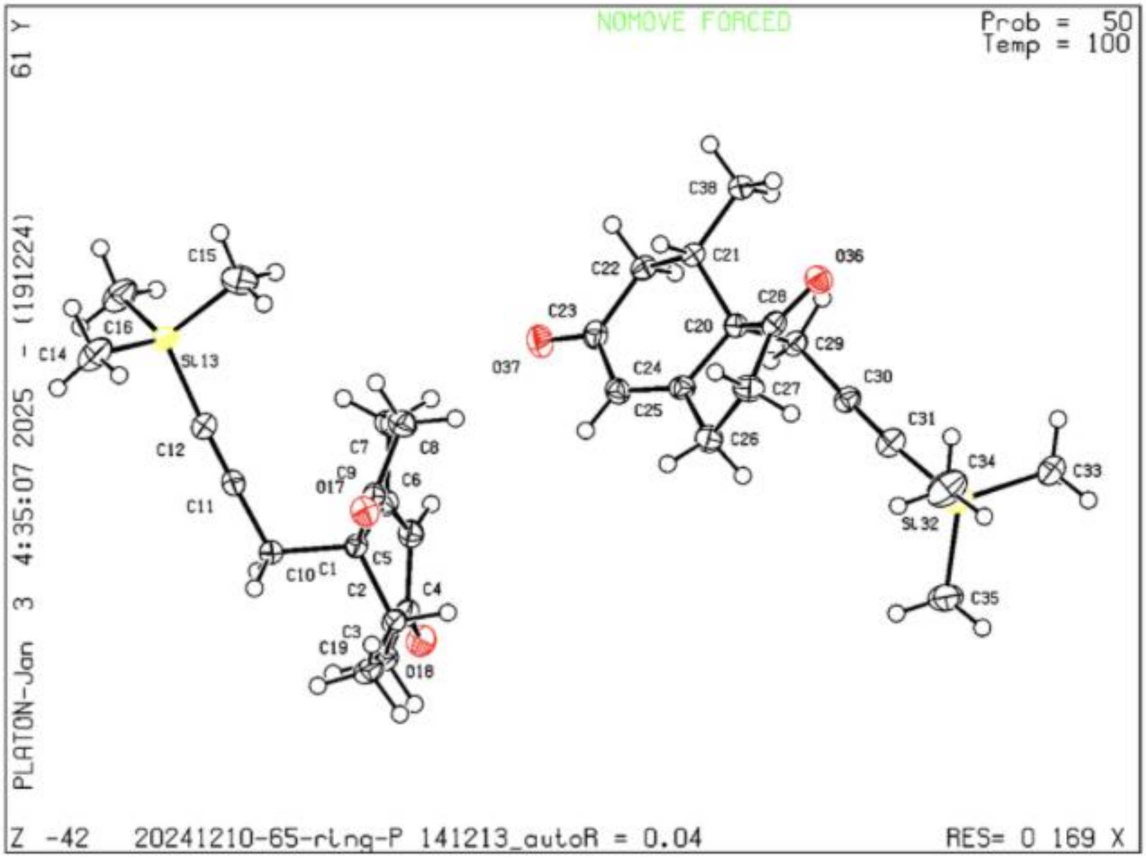

7 (CCDC 2414356)

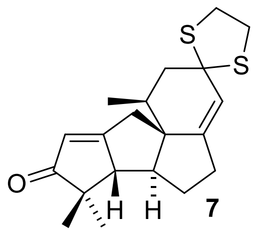

7

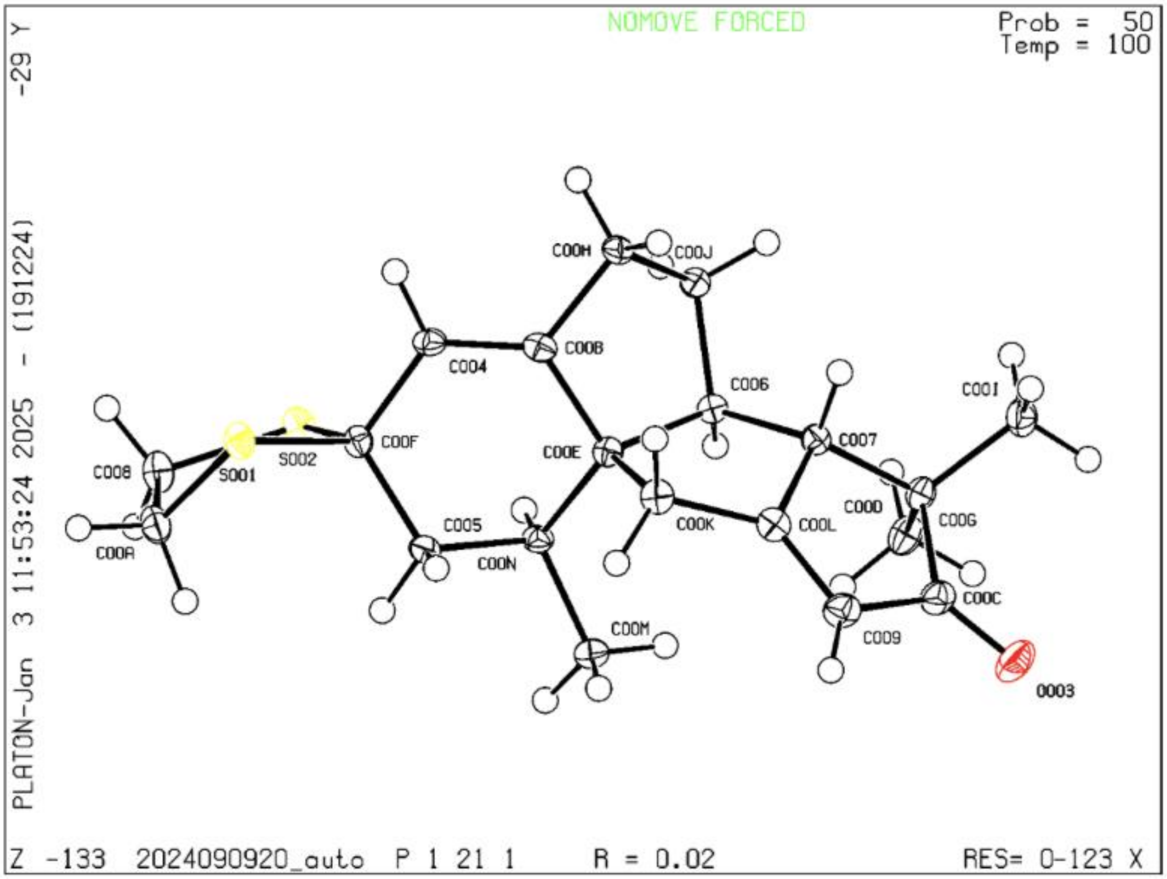

15 (CCDC 2415768)

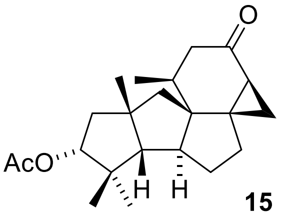

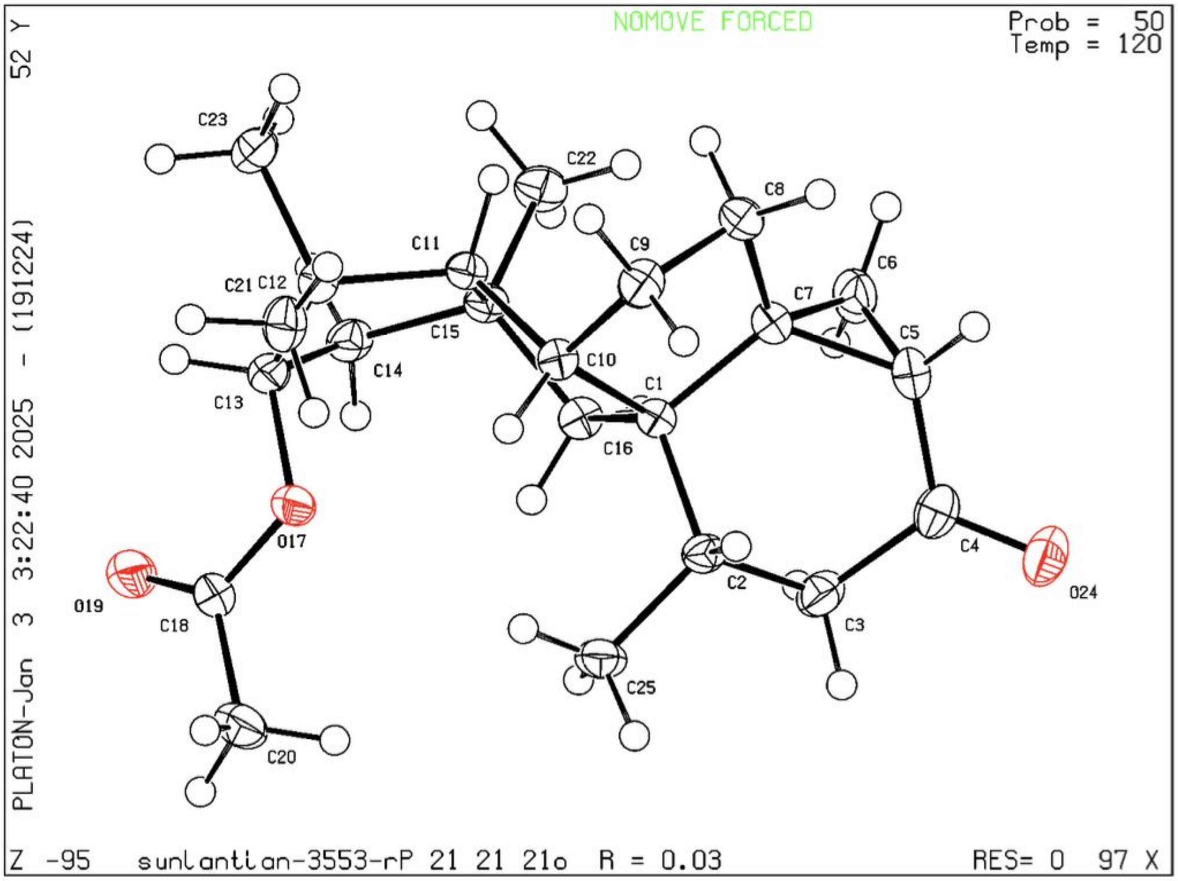

Table S3. Summary of crystal data and refinement parameters for compounds **5**, **7** and **15**

	**5**	**7**	**15**
Formula	$C_{16}H_{22}O_2Si$	$C_{20}H_{26}OS_2$	$C_{22}H_{32}O_3$
$D_{calc.}$/ g cm^{-3}	1.143	1.255	1.209
m/mm^{-1}	1.262	2.629	0.615
Formula Weight	274.14	346.561	344.47
Colour	colourless	colorless	colorless
Shape	block	block	block
Size/mm^3	0.10×0.10×0.10	0.10 ×0.10 ×0.10	0.10 ×0.10 ×0.10
T/K	100.2(7)	99.98(10)	117(20)
Crystal System	triclinic	monoclinic	orthorhombic
Flack Parameter	0.01(3)	-0.006(3)	0.01(8)
Hooft Parameter	-0.014(5)	-0.006(3)	0.01(8)
Space Group	$P1$	$P2_1$	$P2_1$
a/Å	6.32180(10)	8.8578(1)	7.17835(10)
b/Å	11.0760(2)	10.6815(1)	14.1846(2)
c/Å	12.3239(2)	9.7277(1)	18.5883(2)
a/°	67.656(2)	90	90
b/°	87.221(2)	94.572(1)	90
g/°	89.278(2)	90	90
V/Å^3	797.18(3)	917.454(16)	1892.70(4)
Z	1	2	4
Z'	1	1	1
Wavelength/Å	1.54184	1.54184	1.54184
Radiation type	Cu K_a	Cu K_a	Cu K_a
Q_{min}/°	3.882	4.56	7.84
Q_{max}/°	77.042	77.00	153.958
Measured Refl.	21270	10756	10413
Independent Refl.	5944	3691	3787
Reflections with I > 2(I)	5906	3683	3649
R_{int}	0.0312	0.0140	0.0246
Parameters	351	211	231
Restraints	3	1	0
Largest Peak	0.381	0.1758	0.20
Deepest Hole	-0.298	-0.1301	-0.15
GooF	1.080	1.0354	1.054
wR_2 (all data)	0.1186	0.0537	0.0827
wR_2	0.1184	0.0537	0.0817
R_1 (all data)	0.0440	0.0207	0.0327
R_1	0.0438	0.0206	0.0314

IV. HPLC analysis

1. HPLC analysis of (±)-**5**

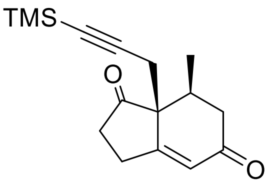

(±)-**5**

Condition: Daicel Chiralcel IC-3, *n*-hexane/isopropanol 95/5, flow rate = 0.5 mL/min, l = 254 nm, t_1 = 31.588 min, t_2 = 36.884 min, t_3 = 43.125, t_4 = 47.755.

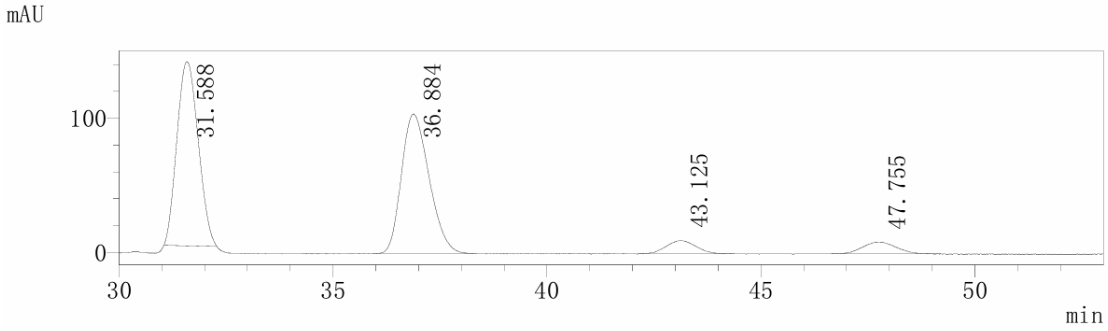

Peak Table

PDA Ch1 254nm

Peak#	Ret. Time	Area	Area%
1	31.588	4750407	45.578
2	36.884	4694408	45.040
3	43.125	487103	4.673
4	47.755	490734	4.708
总计		10422652	100.000

Diastereoselectivity: dr = (45.578 + 45.040)/(4.673 + 4.708) = 1:10

2. HPLC analysis of **5**

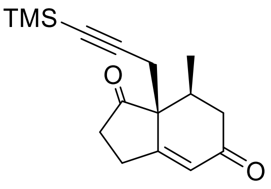

5

Condition: Daicel Chiralcel IC-3, *n*-hexane/isopropanol 95/5, flow rate = 0.5 mL/min, l = 254 nm, t_1 = 31.987 min, t_2 = 37.857 min, t_3 = 43.809, t_4 = 48.627.

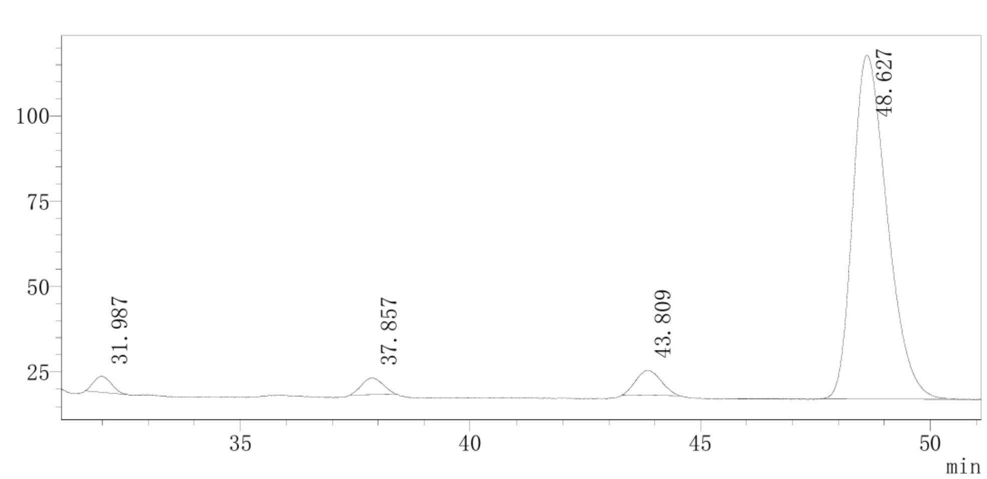

Peak Table

PDA Ch1 254nm

Peak#	Ret. Time	Area	Area%
1	31.987	127483	2.180
2	37.857	159743	2.732
3	43.809	277711	4.750
4	48.627	5281934	90.338
总计		5846871	100.000

Enantioselecivity : %ee = (90.338% - 4.75%)/(90.338% + 4.75%) = 90;

Diastereoseelctivity: dr = (90.338% + 4.723)/(2.180% + 2.732%) = 19:1

V. DFT calculations

Computational details: The molecular geometries of the complexes were optimized using density functional theory (DFT) at the M06-2X/6-31G(d) level.[3,4] Frequency calculations at the same theoretical level were performed to confirm the nature of stationary points: minima (no imaginary frequencies) and transition states (one imaginary frequency), as well as to compute the thermal relative Gibbs free energy correction. The single point energy calculations based on the optimized geometries at M06-2X/6-31G(d) level at a larger basis set def2-TZVP for all the atoms.[5,6] For the purpose of discussion, the Gibbs free energy was obtained from the addition of single point energy at M06-2X/def2-TZVP and thermal correction to Gibbs free energy at M06-2X/6-31G(d) level. Solvent effects of *N*,*N*-dimethylformamide (DMF) were incorporated via the polarizable continuum model (PCM), applied during geometry optimizations, frequency calculations and single point energy calculations.[7] All computations were executed using the Gaussian 16 software package.[8]

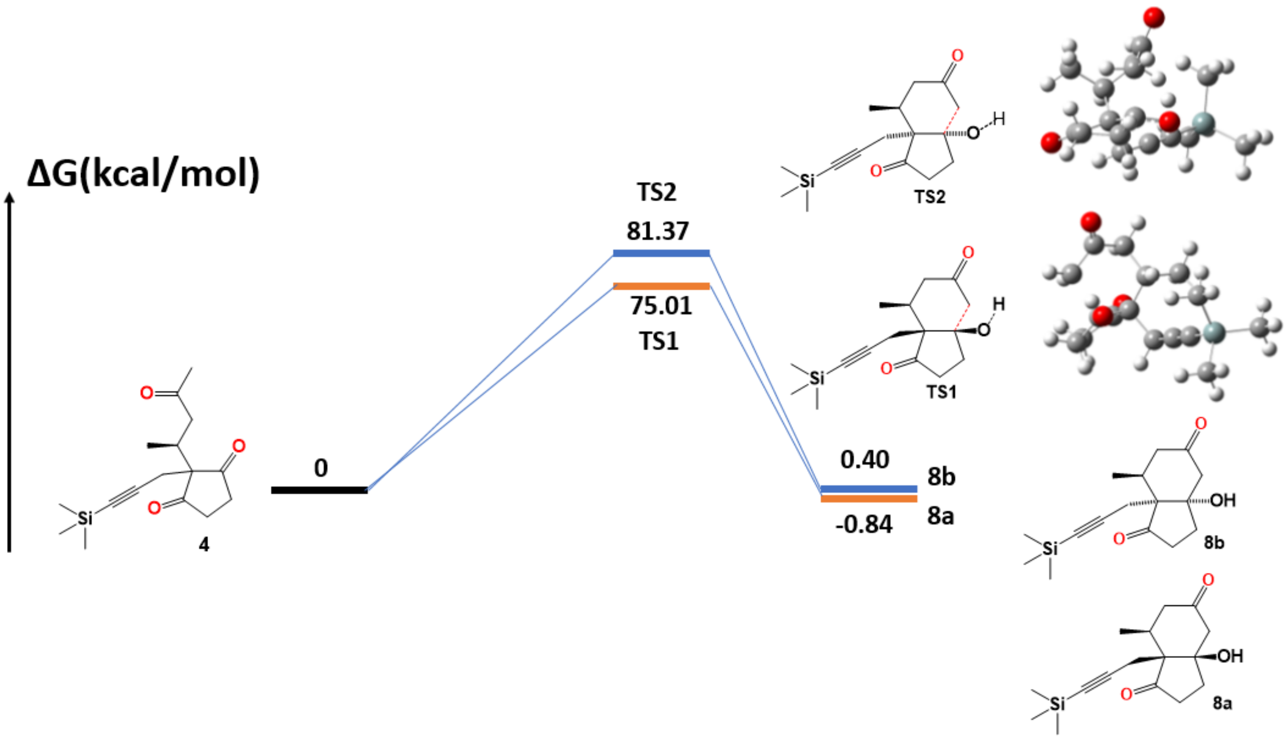

Figure S1. Gibbs energy profile for intramolecular aldol cyclization of **4**.

Table S4. Calculated imaginary frequencies of all transition states species.

Species	Frequencies
**TS1**	-2219.75
**TS2**	-2074.68

Table S5. Calculated energy values of all species at M06-2X/6-31G(d).

Species	Electronic energy (Hartree)	Thermal correction to Gibbs Free Energy (Hartree)	Sum of electronic and zero-point Energies (Hartree)	Sum of electronic and thermal Energies (Hartree)	Sum of electronic and thermal Enthalpies (Hartree)	Sum of electronic and thermal Free Energies (Hartree)
**4**	-1138.906843	0.311000	-1138.537646	-1138.512803	-1138.511859	-1138.595842
**TS1**	-1138.778080	0.309890	-1138.413910	-1138.390559	-1138.389615	-1138.468190
**8a**	-1138.912846	0.319837	-1138.539955	-1138.516870	-1138.515926	-1138.593009
**TS2**	-1138.769065	0.311081	-1138.404754	-1138.381652	-1138.380707	-1138.457984
**8b**	-1138.910620	0.319749	-1138.537994	-1138.515012	-1138.514067	-1138.590871

Table S6. Calculated energy values of all species at M06-2X/def2-TZVP

Species	Electronic energy (Hartree)
**4**	-1139.273717
**TS1**	-1139.153074
**8a**	-1139.283889
**TS2**	-1139.144122
**8b**	-1139.281816

Table S7. Coordinates (Å) for the optimized structure of **4**.

C	2.01072100	2.73754600	0.54659600
C	3.09047200	2.07609300	-0.31708200
C	1.09868700	0.54866400	-0.25891600
H	2.27449400	2.75946000	1.60905100
H	1.78135700	3.76380400	0.25034800
H	3.95225200	1.73308600	0.26338700
H	3.46336800	2.71796900	-1.11903700
C	2.15155800	-1.69691800	0.44437200
H	1.78024800	-2.08110100	-0.51221600
H	1.99624000	-2.51138600	1.16661200
C	0.74950600	1.89025800	0.38463700
O	-0.35298900	2.23756200	0.73576300
C	2.43713600	0.84924400	-0.93222600
O	2.89705700	0.19016700	-1.83815500
C	4.40286000	-2.50197400	-0.49656200
H	4.18885600	-2.26426800	-1.54488300
H	5.47595800	-2.42947000	-0.31793300
H	4.05138400	-3.52134500	-0.31227800
C	3.65818600	-1.51250100	0.36085400
O	4.22634100	-0.62250800	0.96413400
C	1.38132200	-0.45964200	0.91798700
H	2.04110900	0.06461700	1.62205300
C	0.10783800	-0.86308200	1.66425800
H	-0.49669100	-1.55447500	1.07143700
H	0.38569900	-1.36463400	2.59633900
H	-0.51270100	0.00380800	1.90469200
C	-1.30232400	-0.06249800	-0.93171500
C	-2.48165900	-0.18474800	-0.66358600
C	0.10218100	0.05988900	-1.32837400
H	0.45671500	-0.90319800	-1.71276000
H	0.15873400	0.74937700	-2.17941600
Si	-4.22922600	-0.33064900	-0.08998700
C	-5.15770500	1.23081600	-0.56594400
H	-5.16875800	1.36944300	-1.65144300
H	-6.19580500	1.17369300	-0.22125800
H	-4.69850900	2.11509100	-0.11337200
C	-5.02021600	-1.83198900	-0.89460300
H	-6.05696300	-1.93790000	-0.55749600
H	-5.02702900	-1.73952300	-1.98503600
H	-4.48398700	-2.74936000	-0.63284600
C	-4.19012000	-0.52877300	1.77808100
H	-3.62551500	-1.42147200	2.06580700
H	-3.71956300	0.33816300	2.25273700
H	-5.20565200	-0.62537700	2.17673700

Table S8. Coordinates (Å) for the optimized structure of **TS1**.

C	2.24421000	1.63279800	1.15432000
H	3.14343500	1.13277500	1.53439600
H	2.00326800	2.46588600	1.81927700
C	1.06257600	0.63672900	1.14613900
H	0.20854800	1.13212600	0.66308100
C	0.67117700	0.30275700	2.58952200
H	0.51530000	1.23140600	3.14529800
H	-0.26116600	-0.26589400	2.63561400
H	1.46158900	-0.26721400	3.08431200
C	2.49015100	2.19061800	-0.23021100
O	2.26522300	3.35826800	-0.50482800
C	2.28458500	-1.51384200	-1.81427200
H	1.55569900	-2.25684800	-2.16094500
H	2.83068500	-1.17288200	-2.69492000
C	3.16397700	-2.04022700	-0.67586900
H	3.11207700	-3.12692700	-0.56136700
H	4.22094800	-1.78298500	-0.79136200
C	1.34330500	-0.61644200	0.29255100
C	2.63409000	-1.38379900	0.59931000
O	3.15034100	-1.47148300	1.68729100
C	0.19909900	-1.68319100	0.45404500
H	0.28861000	-2.09506800	1.46523400
H	0.35830900	-2.52270400	-0.23239600
C	-1.14473000	-1.13864100	0.26617400
C	-2.26799100	-0.68965100	0.14746900
C	2.86332700	1.18161600	-1.24633800
H	3.64989500	0.50379100	-0.91062000
H	1.56787500	0.90369000	-1.79681600
H	3.14671200	1.61252400	-2.21570200
C	1.45762100	-0.39239100	-1.21260700
O	0.51419300	0.20332300	-1.90782200
Si	-3.94853000	0.04788300	-0.09087400
C	-4.82449000	-0.90169300	-1.45260600
H	-5.82321700	-0.48463700	-1.62046900
H	-4.26969400	-0.84200400	-2.39390300
H	-4.93848500	-1.95756600	-1.18882600
C	-4.89660700	-0.08459700	1.52374600
H	-4.38206700	0.44925800	2.32858800
H	-5.89462300	0.35225100	1.41113500
H	-5.01641000	-1.12892000	1.82788200
C	-3.71673400	1.84560400	-0.57956600
H	-3.18500900	2.39995200	0.20005900
H	-3.14362900	1.92904700	-1.50812400
H	-4.68868100	2.32583500	-0.73516200

Table S9. Coordinates (Å) for the optimized structure of **8a**.

C	-2.28707300	-2.22417100	1.16140000
C	-3.37153700	-1.35126200	0.52054100
C	-1.23774800	-0.32171400	-0.07198100
H	-2.33929800	-2.27703200	2.25219400
H	-2.30595700	-3.25001300	0.78212200
H	-3.77572200	-0.61922200	1.22644700
H	-4.21930400	-1.92921700	0.14098600
C	-0.96044400	-1.60010600	0.72997100
O	0.12971100	-2.04923900	0.99292800
C	-0.22663600	-0.05594900	-1.20541000
H	-0.36621000	-0.83477300	-1.96034200
H	-0.48750000	0.89041100	-1.69788200
C	1.19588500	-0.00756100	-0.85390500
C	2.39125000	0.06189800	-0.64150600
C	-3.39336300	0.55614000	-1.20610500
H	-2.87776300	0.89130900	-2.11605800
H	-4.41942400	0.28444300	-1.47363200
C	-2.64342100	-0.65429900	-0.64642500
C	-3.42003600	1.70681800	-0.22255600
O	-4.44329100	2.28957900	0.06992700
C	-0.03831100	1.25939900	1.60484000
H	-0.24213600	1.91551100	2.45641000
H	0.53079500	0.39512900	1.95848400
H	0.58756000	1.80564900	0.89459600
C	-1.36280800	0.83557900	0.96942300
H	-1.99784600	0.46265600	1.78421000
C	-2.07565600	2.06239300	0.37029100
H	-2.21746800	2.82997500	1.13556000
H	-1.45135500	2.49351000	-0.42511800
O	-2.41752500	-1.61467300	-1.67124200
H	-3.28017700	-1.94062900	-1.97275100
Si	4.16369600	0.17368400	-0.14887900
C	5.07009000	1.29922700	-1.34926400
H	4.63685200	2.30420700	-1.35299700
H	6.12381500	1.38591600	-1.06300600
H	5.02837400	0.90581200	-2.36965100
C	4.91855100	-1.54613300	-0.17482100
H	4.87739100	-1.98133300	-1.17812000
H	5.96896200	-1.50318200	0.13278000
H	4.39198700	-2.21769400	0.51027300
C	4.22205600	0.88746500	1.58884200
H	3.76250000	1.88061200	1.62006500
H	3.68660200	0.24349300	2.29377600
H	5.25762200	0.98119600	1.93305700

Table S10. Coordinates (Å) for the optimized structure of **TS2**

C	3.02575300	-0.76255000	-1.77332500
H	2.67936200	-1.62827300	-2.35265700
H	3.54442100	-0.09487400	-2.46286100
C	3.83485600	-1.20894000	-0.55978400
H	4.45506300	-2.09151300	-0.72395000
H	4.49078800	-0.41037000	-0.18823700
C	1.47246100	-0.73212400	0.17346000
C	2.77666100	-1.48863700	0.49381300
O	2.89706700	-2.24266300	1.42959100
C	0.38290400	-1.83091900	-0.02885300
H	0.48059400	-2.51492700	0.82243500
H	0.59383800	-2.41146300	-0.93454200
C	-0.98809500	-1.32356800	-0.07860500
C	-2.13465900	-0.91827500	-0.06610400
H	1.46314500	1.13661200	-1.68868600
C	1.74541500	-0.14078400	-1.22707900
O	0.74713000	0.09734800	-2.06614900
Si	-3.83966600	-0.20450300	-0.03269200
C	-4.61113200	-0.39132700	-1.73382200
H	-5.62041000	0.03416000	-1.73820800
H	-4.02055300	0.12897600	-2.49400400
H	-4.68652600	-1.44463400	-2.02078100
C	-4.85338900	-1.12132900	1.25451300
H	-4.40494400	-1.02937000	2.24848200
H	-5.86675600	-0.70823500	1.30014400
H	-4.93223600	-2.18555700	1.01219300
C	-3.67669700	1.60976400	0.42638000
H	-3.19244000	1.72589800	1.40130600
H	-3.08143100	2.15151900	-0.31559000
H	-4.66403300	2.08089800	0.47901400
C	1.04801900	0.23992500	1.32985000
H	0.27844700	-0.29724000	1.89428600
C	2.15651700	0.59462200	2.33034600
H	2.51695800	-0.28979500	2.85711000
H	3.01892900	1.08437200	1.86450700
H	1.75209000	1.29016500	3.07157500
C	0.37698900	1.51452800	0.79688600
H	-0.08679300	2.07325000	1.61488700
H	-0.41895900	1.24055000	0.08916000
C	1.30325900	2.43888200	0.03800500
O	1.15772300	3.64418500	0.02473100
C	2.32845300	1.73858000	-0.80911700
H	3.14535500	1.34157300	-0.20440700
H	2.77217700	2.38326700	-1.57825400

Table S11. Coordinates (Å) for the optimized structure of **8b**.

C	-2.91311900	-0.72231400	1.81358000
H	-2.41912700	-1.53444800	2.35758300
H	-3.48199800	-0.12956300	2.53554900
C	-3.76653000	-1.28631100	0.67855300
H	-4.29043900	-2.21608100	0.90827900
H	-4.51612600	-0.56041900	0.33649900
C	-1.48783600	-0.67280800	-0.18644700
C	-2.76728000	-1.49236900	-0.44500100
O	-2.90726500	-2.24033400	-1.38508100
C	-0.39772300	-1.75397200	0.07893900
H	-0.49117900	-2.49568900	-0.72354800
H	-0.60513400	-2.27085600	1.02268600
C	0.98140000	-1.26299500	0.09054600
C	2.13560400	-0.88045800	0.05823400
C	-1.82941900	0.11993800	1.11233800
Si	3.84973300	-0.19502900	0.02408000
C	4.70978300	-0.63332800	1.63483700
H	5.72762100	-0.22878700	1.64167600
H	4.17411000	-0.21755400	2.49373000
H	4.77577100	-1.71781400	1.76569100
C	4.77603600	-0.93160900	-1.43406800
H	4.27876900	-0.69182600	-2.37899200
H	5.79414400	-0.52974000	-1.47558000
H	4.84508700	-2.02058800	-1.35099900
C	3.71067400	1.66930600	-0.16343000
H	3.19239600	1.93310700	-1.09098500
H	3.15571100	2.10791000	0.67199500
H	4.70554700	2.12679200	-0.18827900
C	-1.07733200	0.21977700	-1.40096800
H	-0.31487000	-0.33131400	-1.96267700
C	-2.20643400	0.55337500	-2.38510200
H	-2.58562200	-0.34449200	-2.87501800
H	-3.05337500	1.05471600	-1.90284200
H	-1.82410600	1.22835100	-3.15684400
C	-0.41809200	1.50981100	-0.88674500
H	-0.06078000	2.13390300	-1.71114000
H	0.44357200	1.25560400	-0.25586900
C	-1.35540600	2.32362400	-0.02549300
O	-1.31397500	3.53552600	0.02983500
C	-2.35449500	1.52320600	0.78999100
H	-3.29548600	1.47454600	0.22658000
H	-2.57399000	2.06916600	1.71401100
O	-0.65501100	0.24130300	1.89766600
H	-0.90573700	0.64626800	2.74340800

VI. General Information for *in vitro* assays

***Cell lines**:* A549, RAW264.7, and HL-60 cell lines were purchased from ATCC.

***Cytotoxicity Assessment in vitro**:* To evaluate the biological activity of synthetic leptosphin C (**1**), CCK8 tests were carried out. Take RAW264.7 (Mouse macrophage cell line) for example, 9000 RAW264.7 cells per well were pre-plated in 96-well plates. After 24 h, the indicated formulations were added and incubated with cells for another 24 h. Then the CCK8 solution (10 μL) was added into each well. After 1 – 4 h incubation at 37 °C, the absorbance at 450 nm was recorded by a microplate reader (BioTek H1, USA). The biological activity of leptosphin C (**1**) to A549 cells (human lung adenocarcinoma cell line), and HL-60 cells (human promyelocytic leukemia cell line) were tested by the same strategy as described above. The data were presented as mean ± SEM (n = 3) from three independent experiments. Data were analyzed by one-way ANOVA with Turkey multiple comparisons post-test (*P < 0.05, **P < 0.01, ***P < 0.001, ****P < 0.0001).

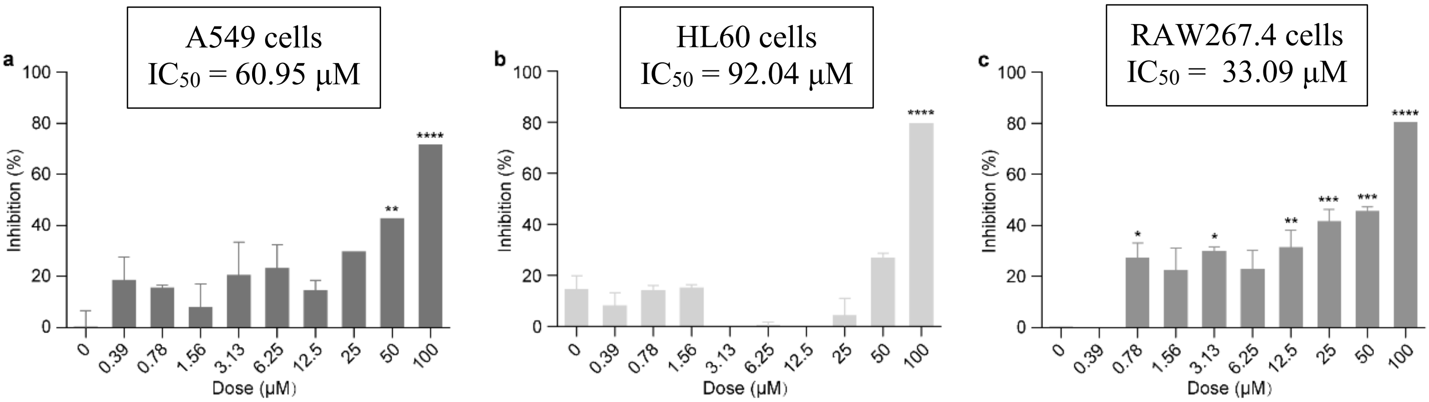

Figure S2. CCK8 assays of synthetic leptosphin C (**1**)

VII. NMR Spectra of new Compounds

^{1}H (400 MHz) and ^{13}C{^{1}H} (100 MHz) NMR spectra of **5** in $CDCl_3$

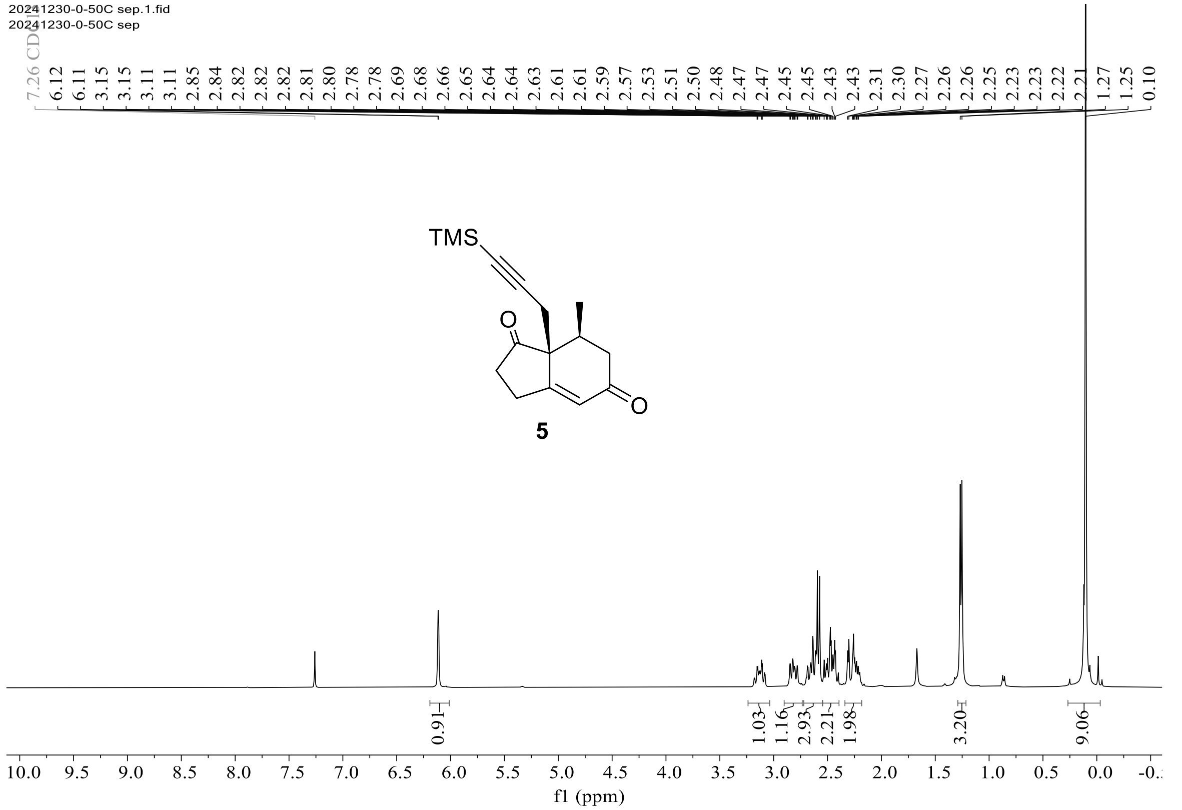

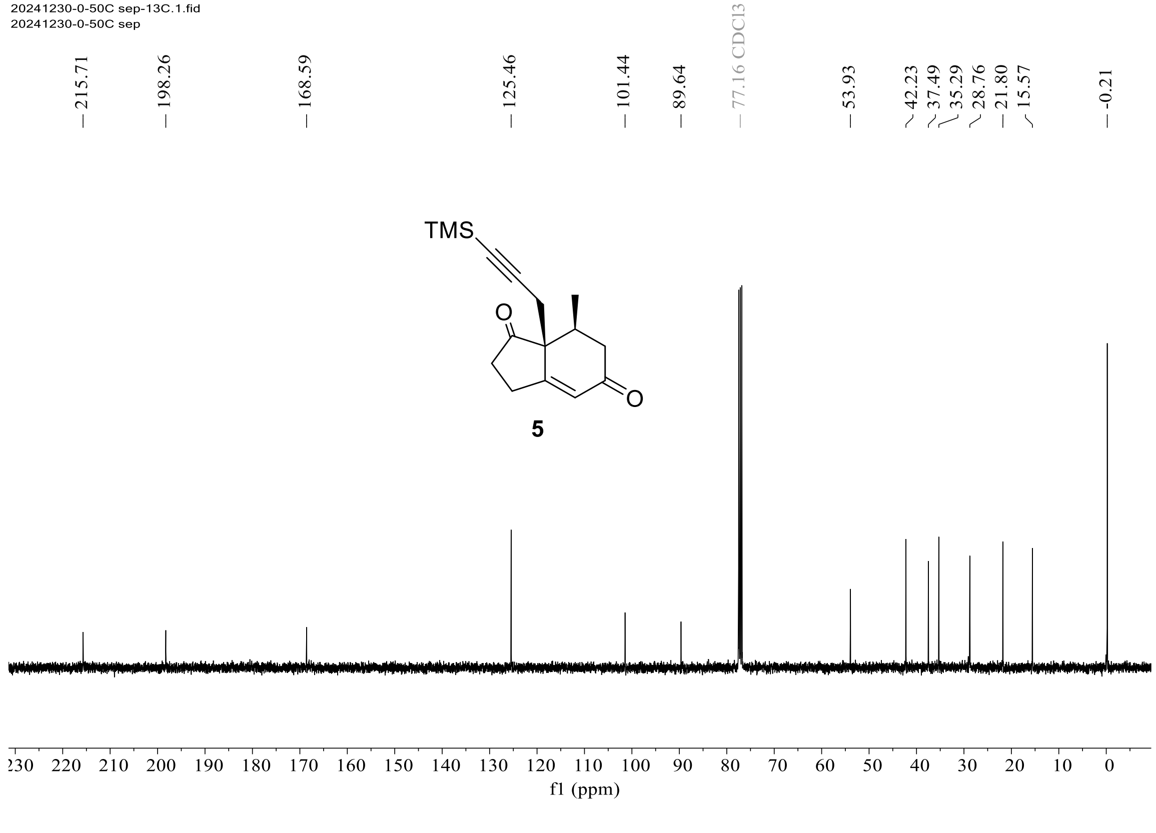

^1H (400 MHz) and $^{13}C\{^1H\}$ (100 MHz) NMR spectra of **9** in $CDCl_3$

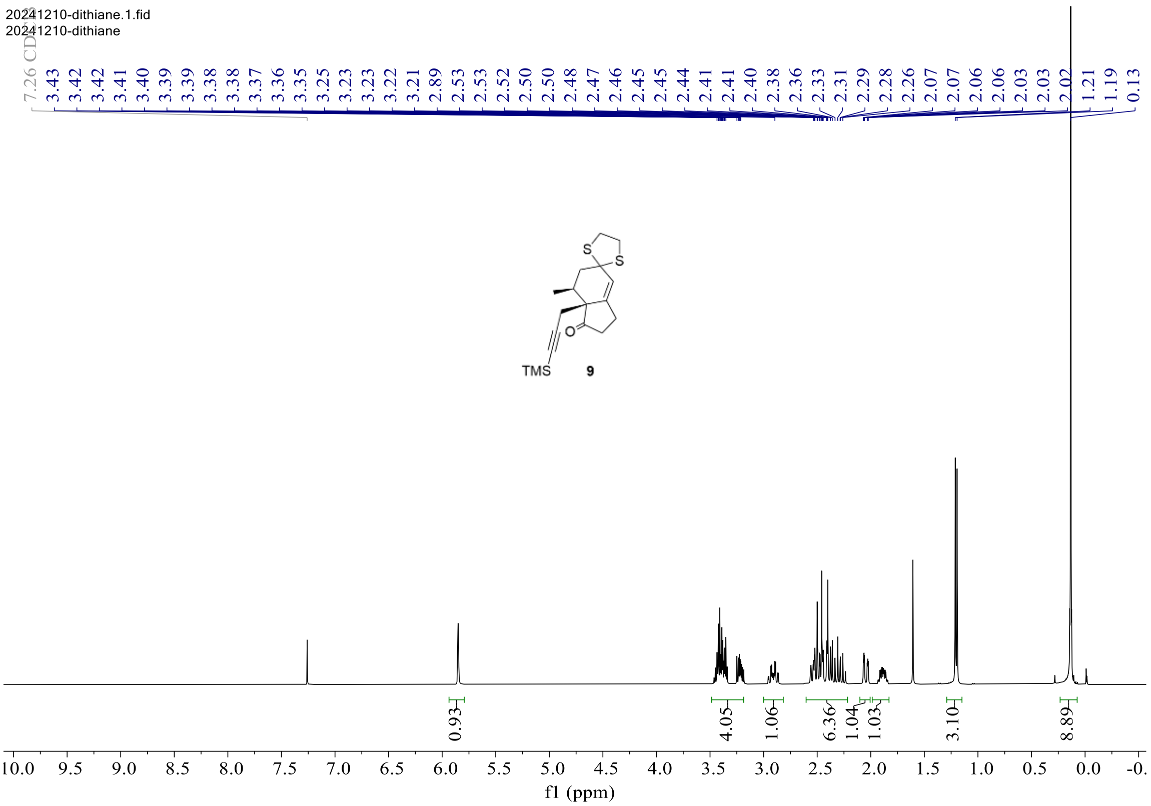

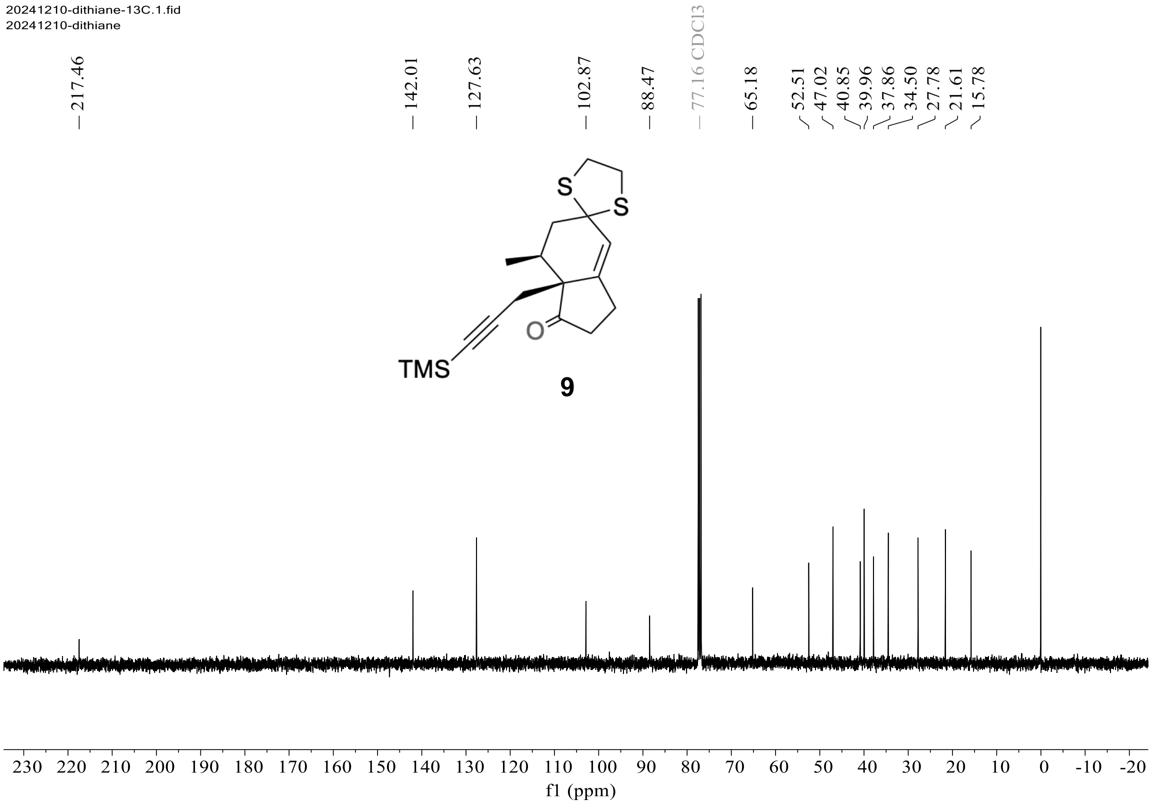

^{1}H (400 MHz) and ^{13}C{^{1}H} (100 MHz) NMR spectra of **10** in $CDCl_3$

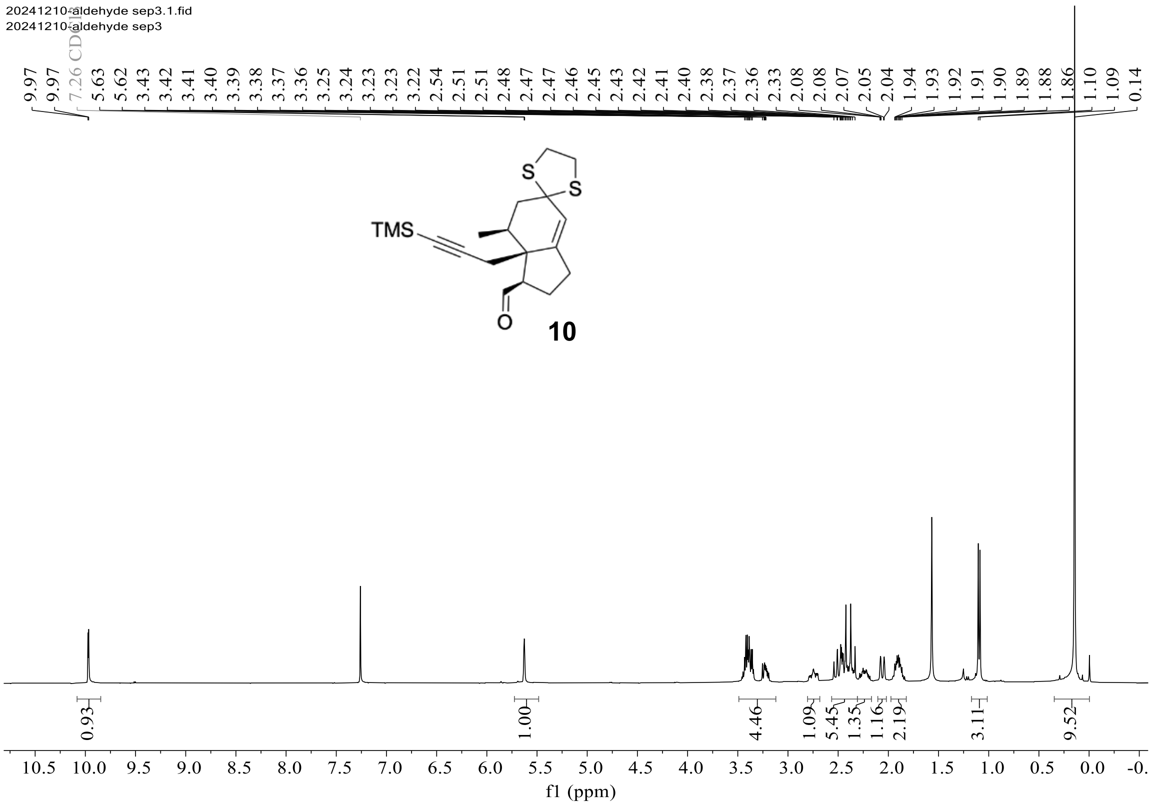

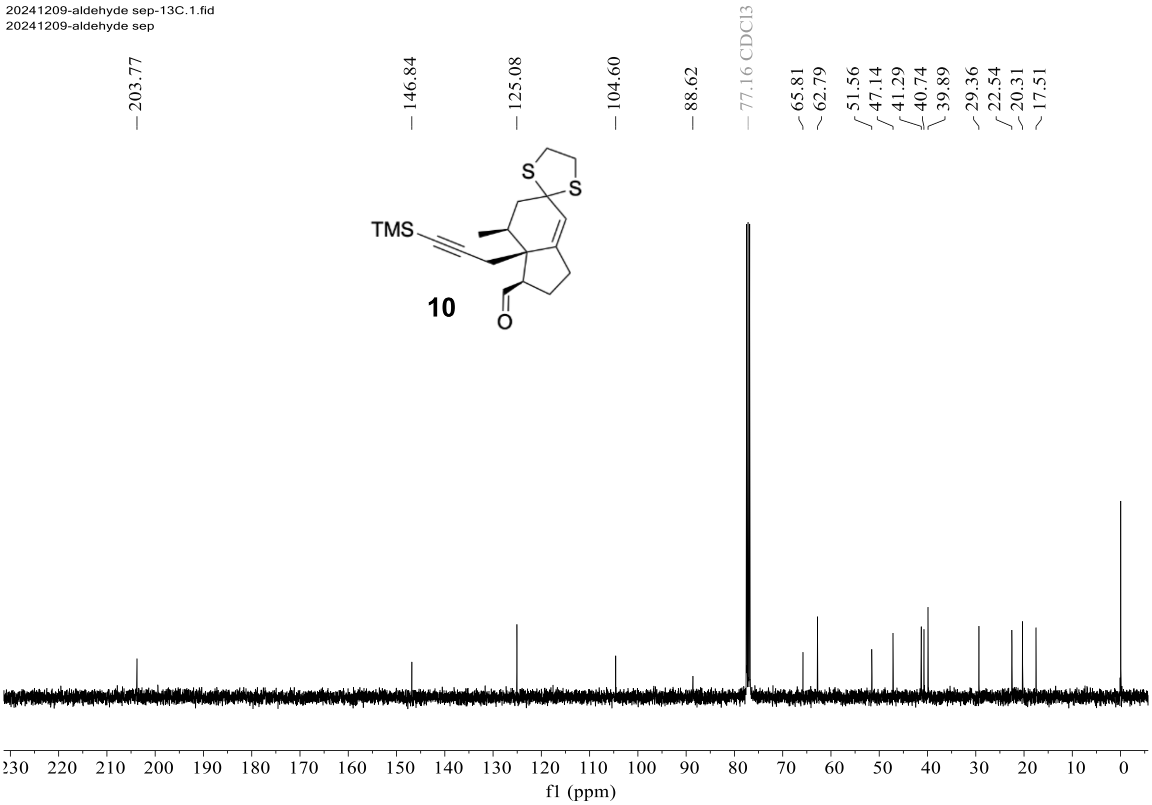

^{1}H (400 MHz) and ^{13}C{^{1}H} (100 MHz) NMR spectra of **6** in $CDCl_3$

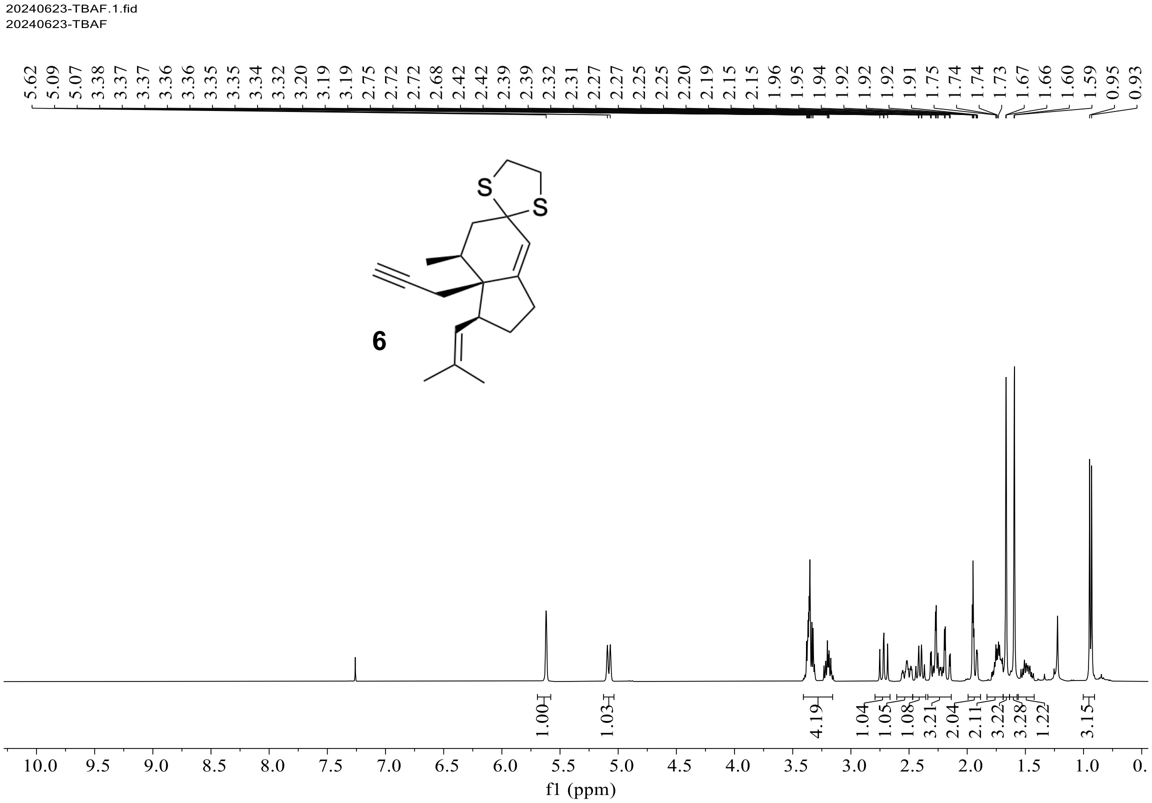

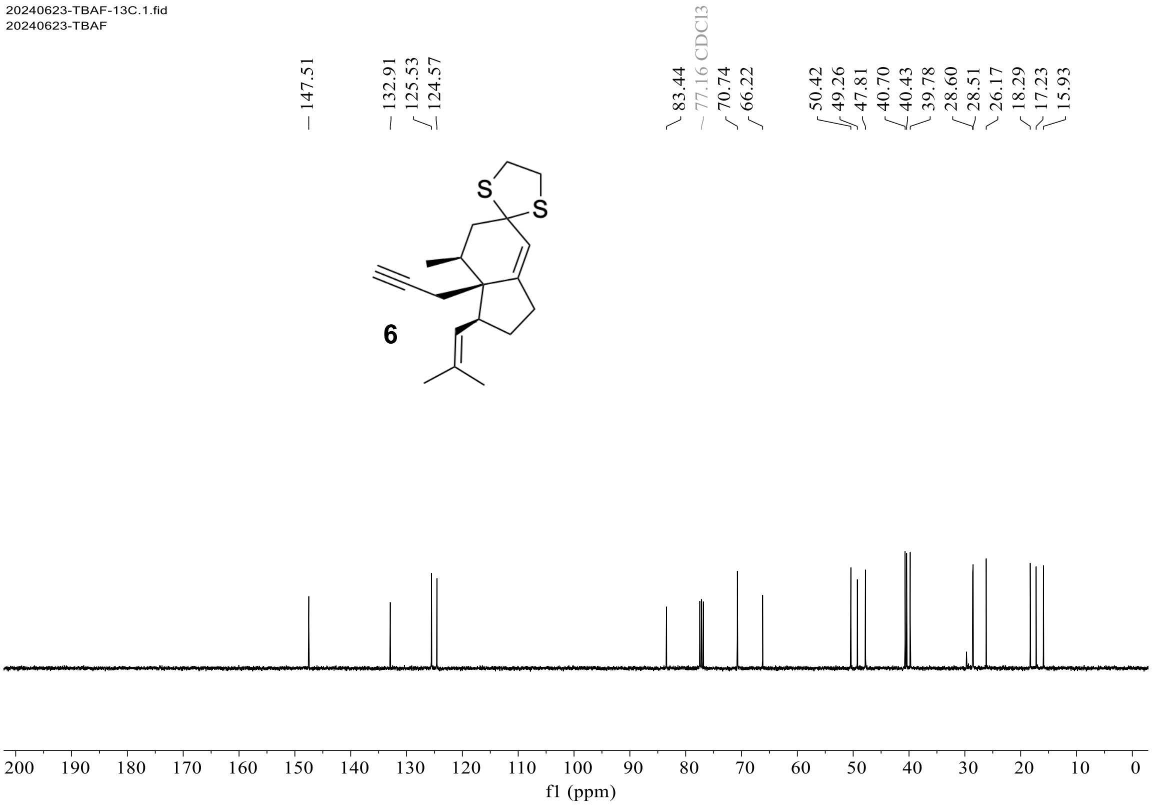

^{1}H (400 MHz) and ^{13}C{^{1}H} (100 MHz) NMR spectra of **7** in $CDCl_3$

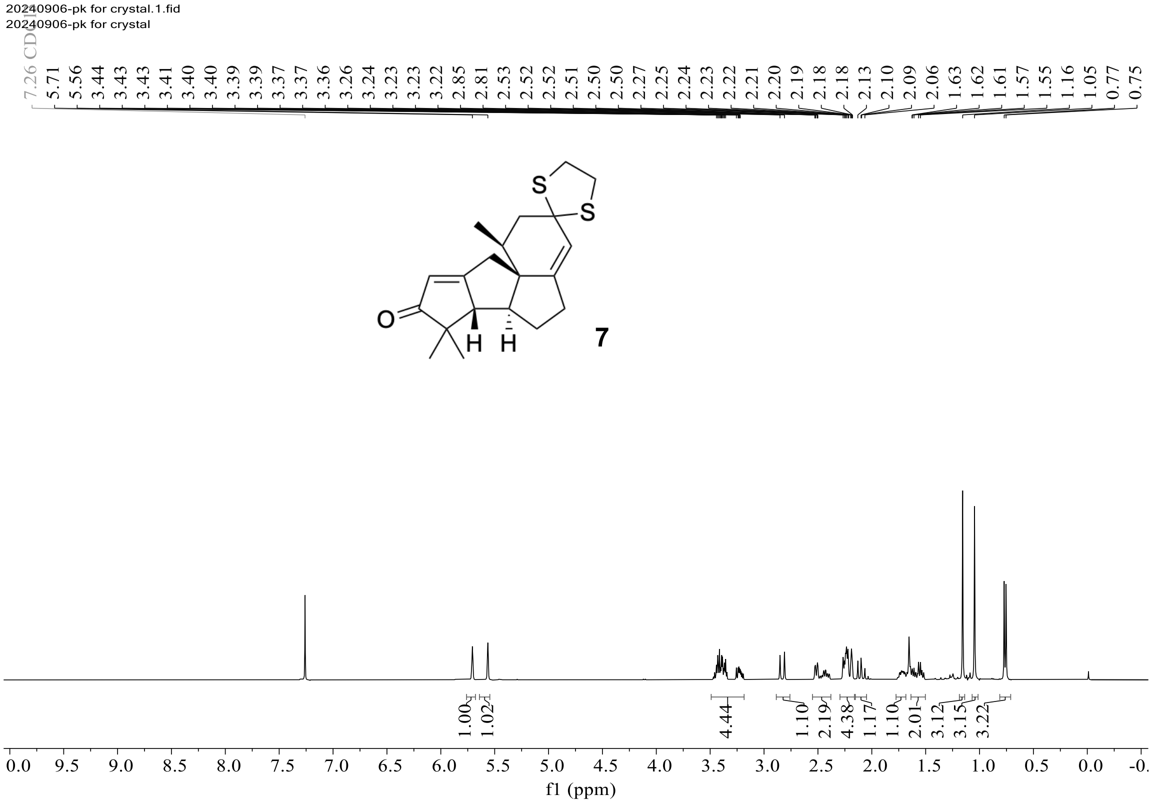

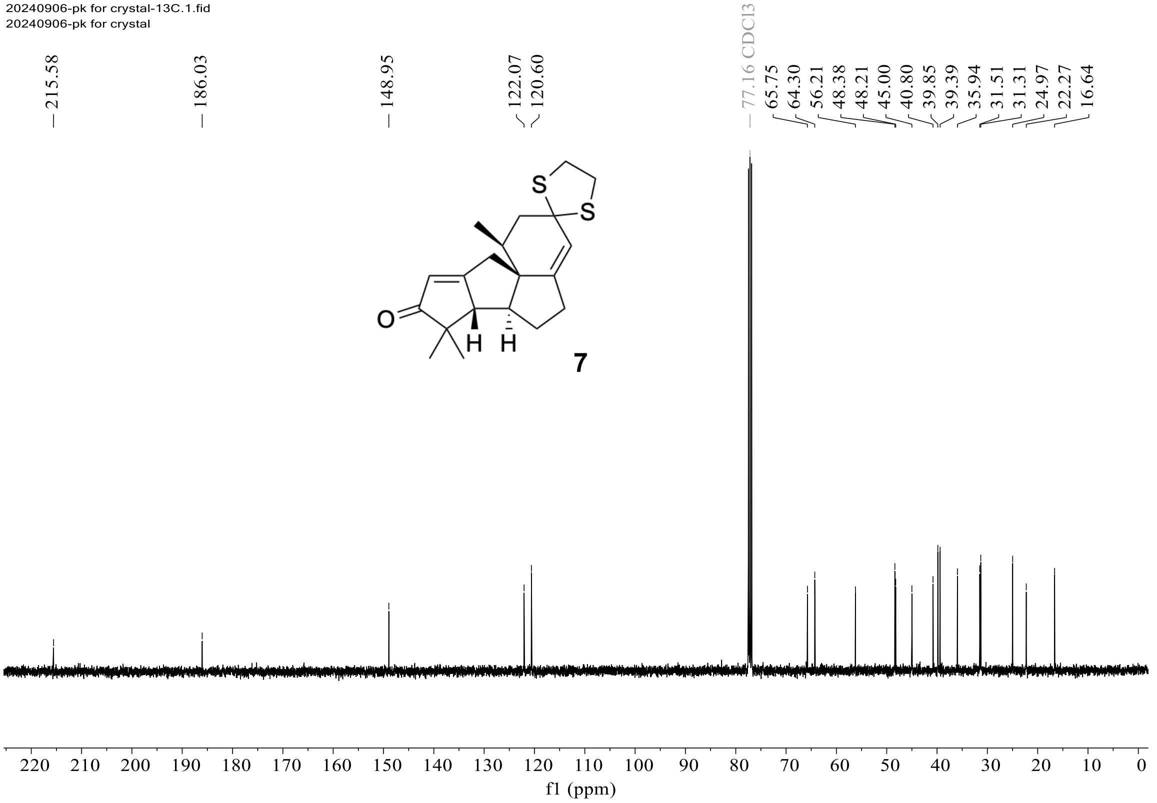

^{1}H (400 MHz) and $^{13}C\{^{1}H\}$ (100 MHz) NMR spectra of **11** in $CDCl_3$

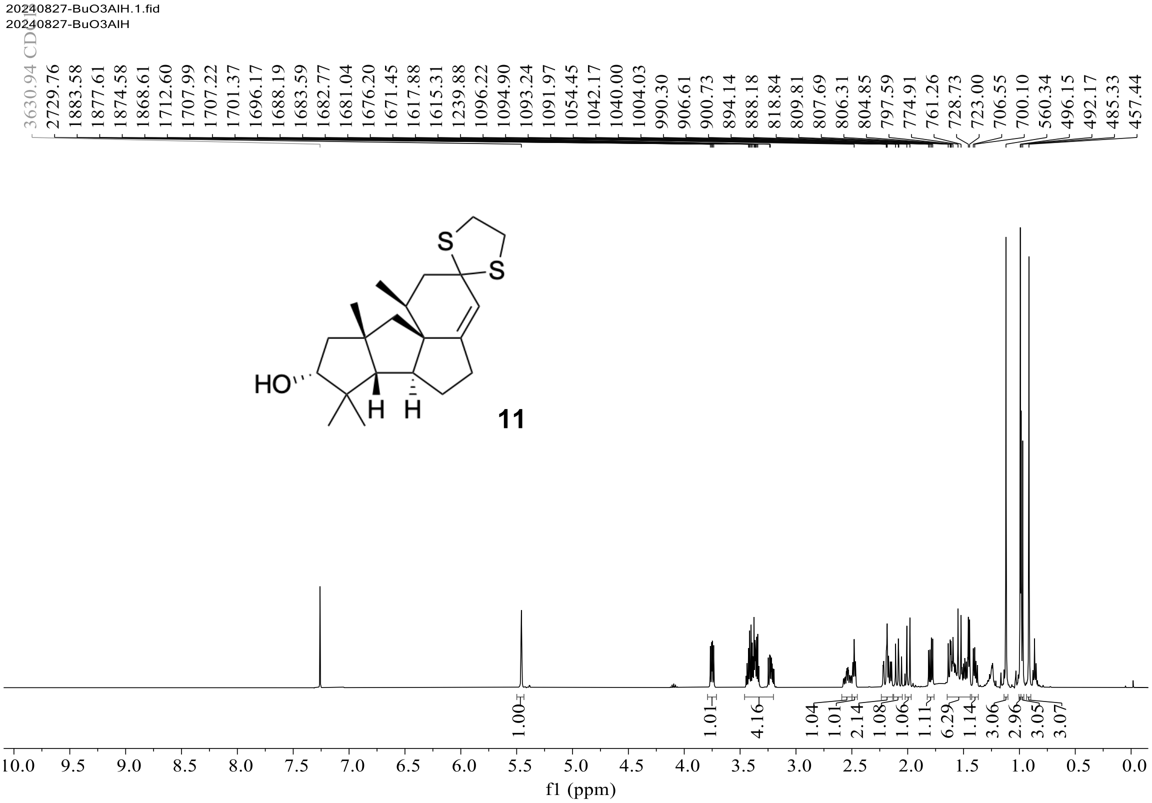

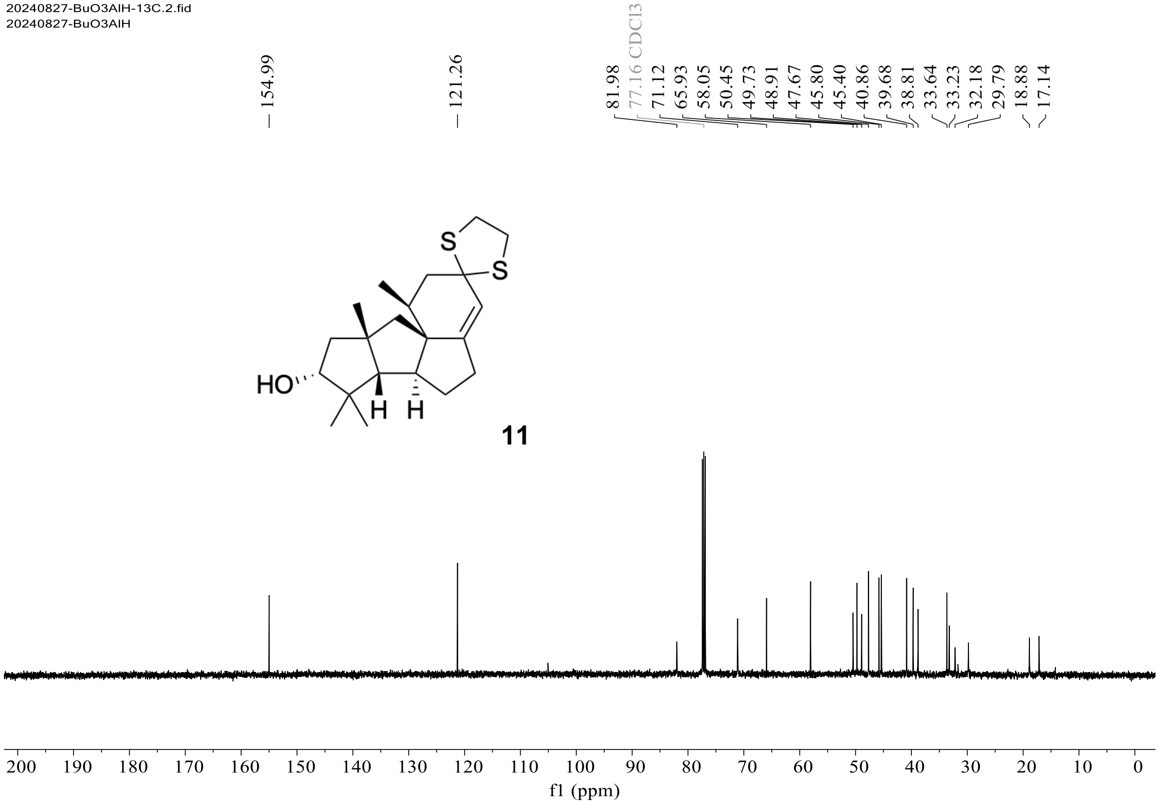

^{1}H (400 MHz) and $^{13}C\{^{1}H\}$ (100 MHz) NMR spectrum of **12** in $CDCl_3$

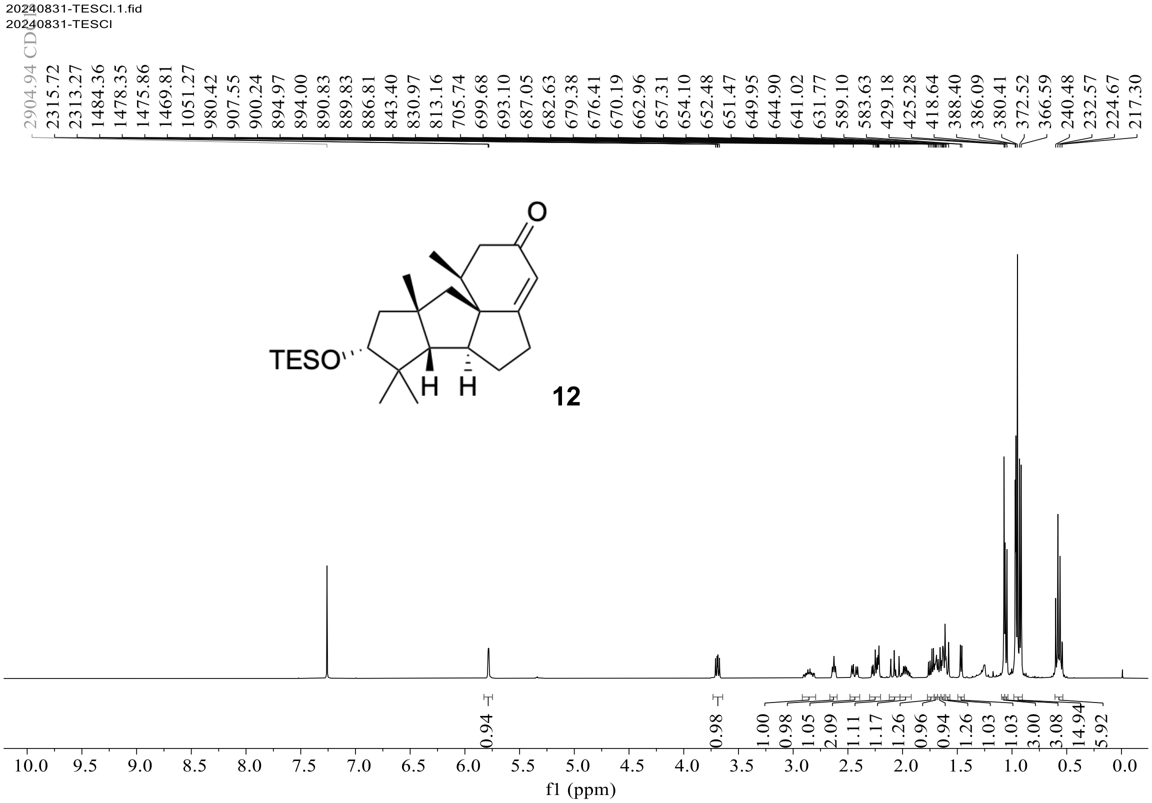

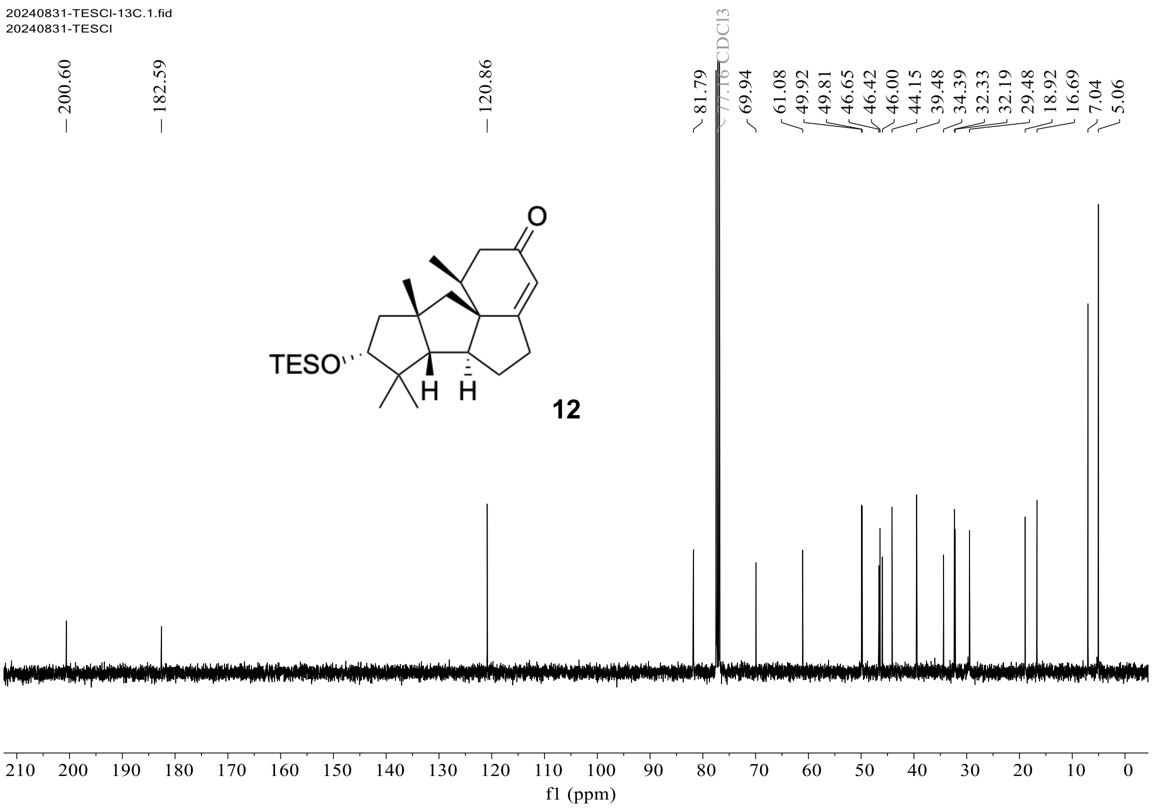

^{1}H (400 MHz) and $^{13}C\{^{1}H\}$ (100 MHz) NMR spectra of **18** in $CDCl_3$

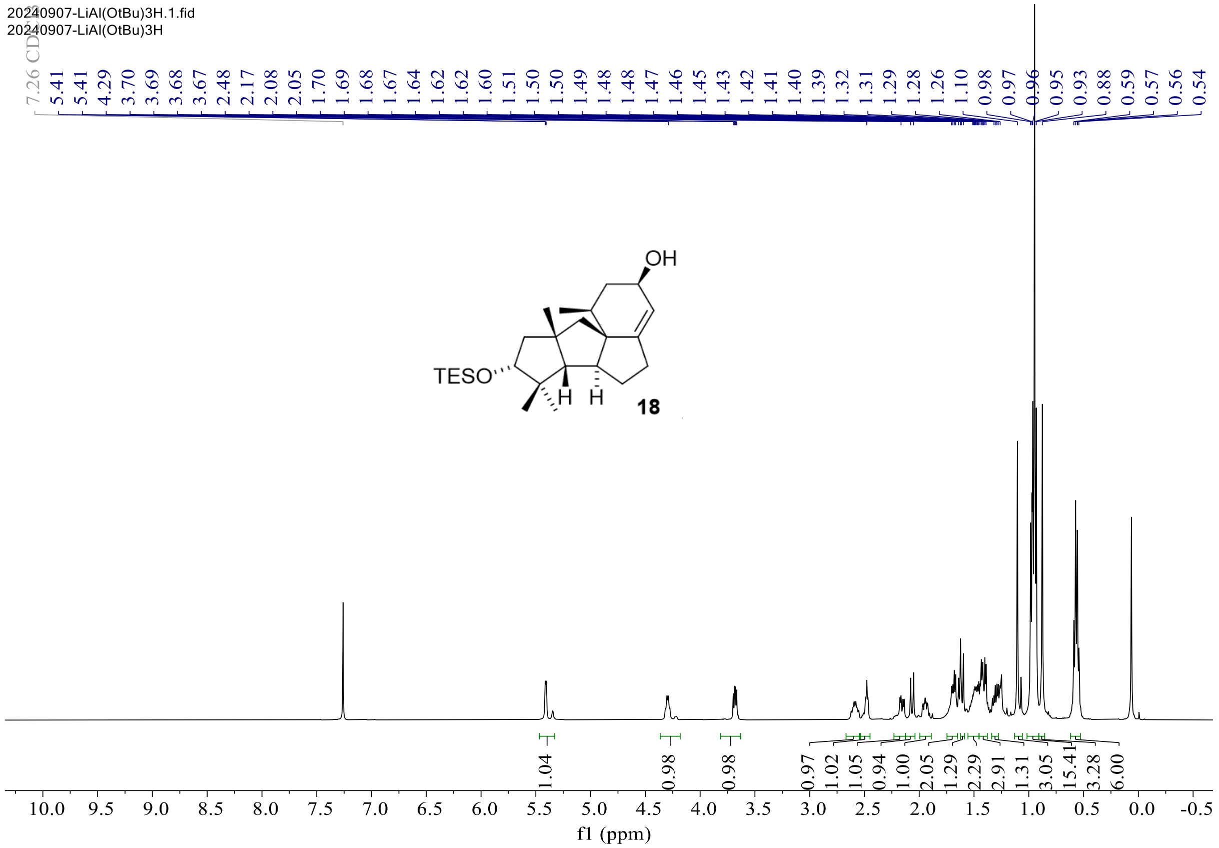

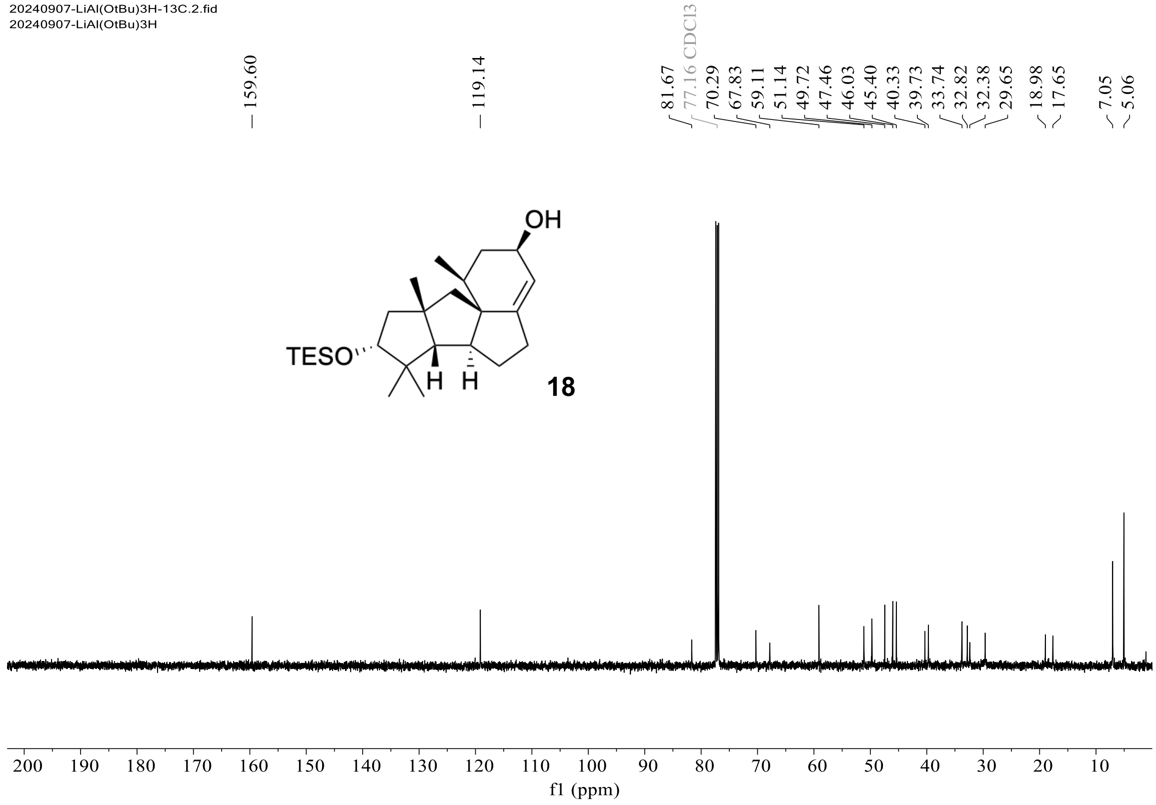

^{1}H (400 MHz) and $^{13}C\{^{1}H\}$ (100 MHz) NMR spectra of **13** in $CDCl_3$

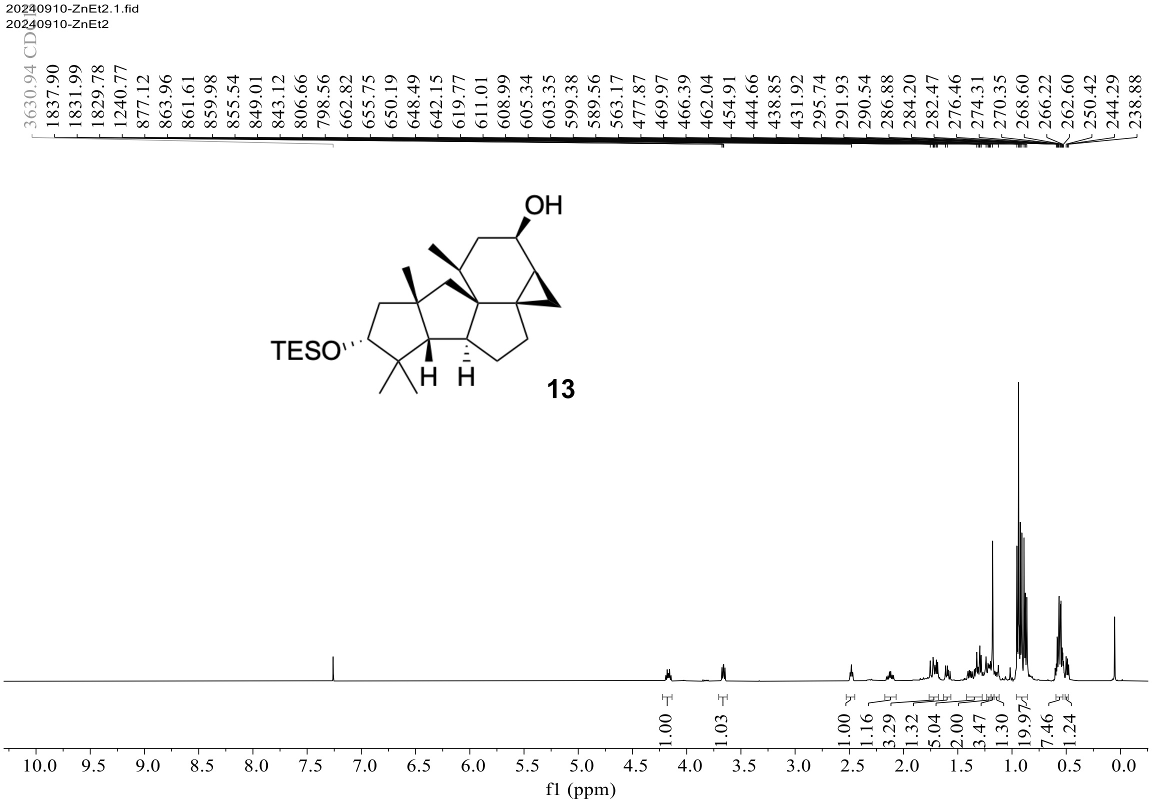

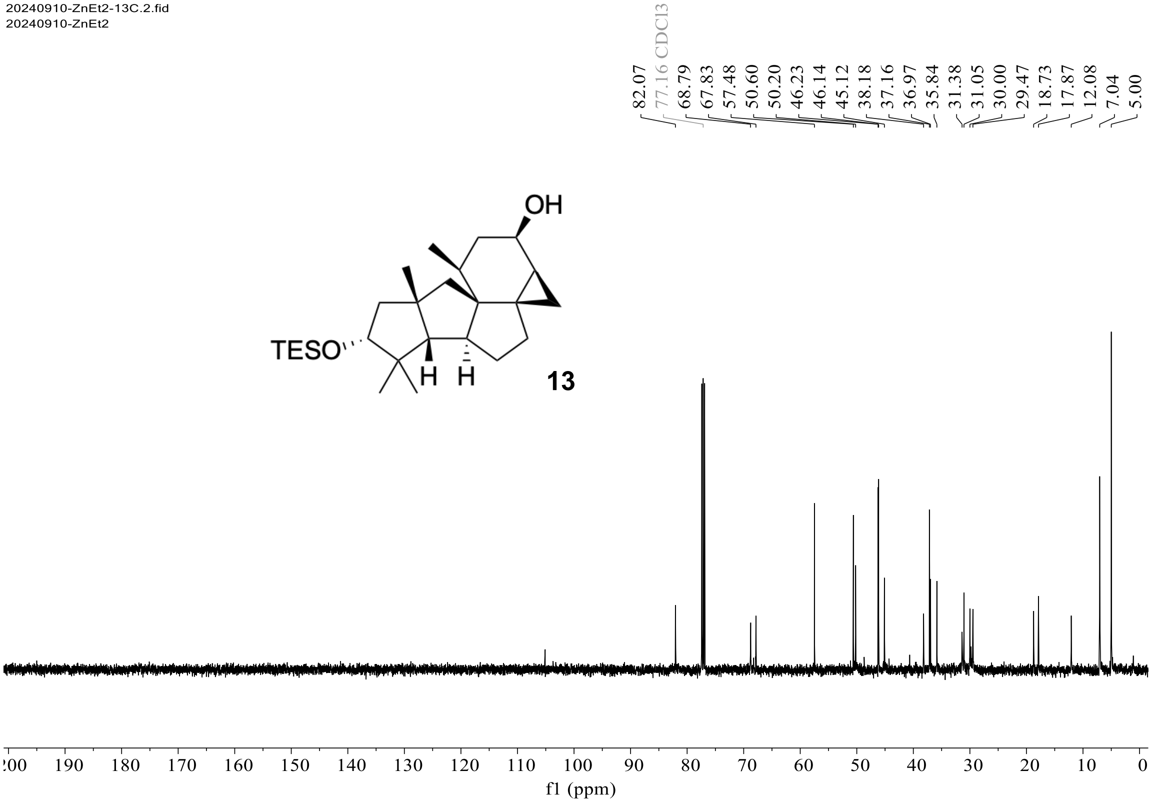

^{1}H (400 MHz) and ^{13}C{^{1}H} (100 MHz) NMR spectra of **14** in $CDCl_3$

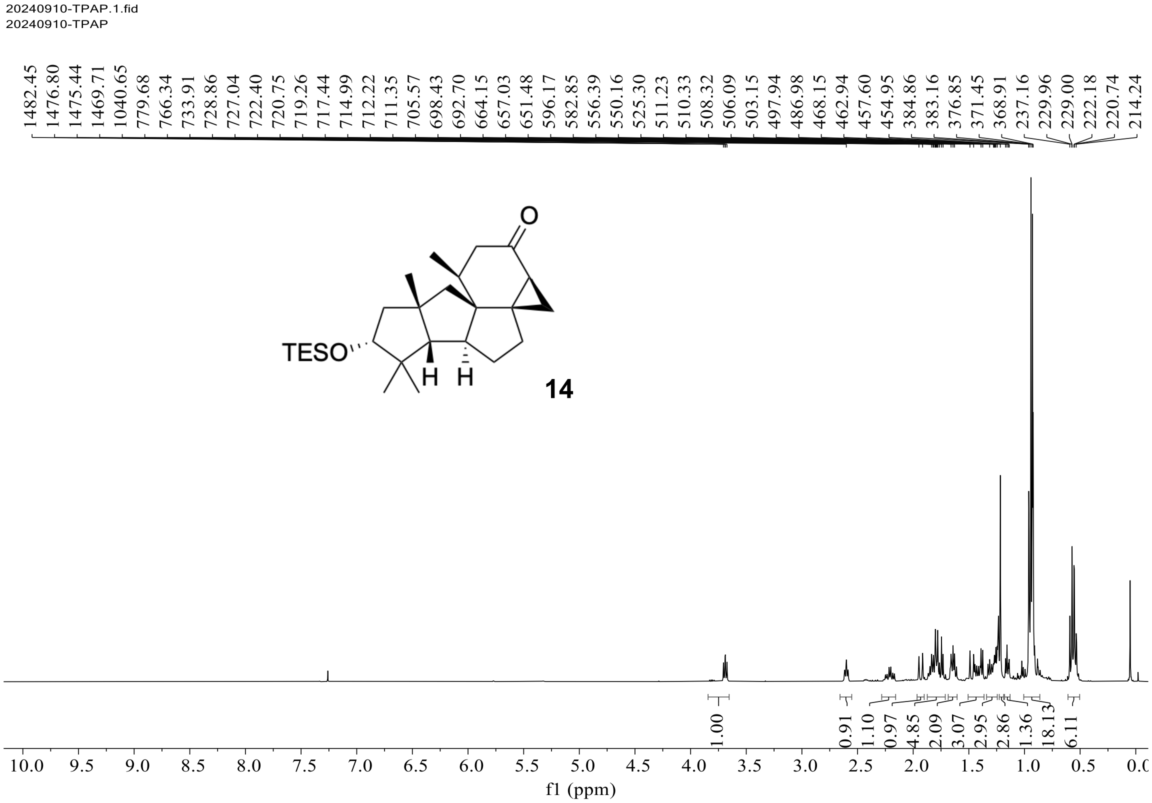

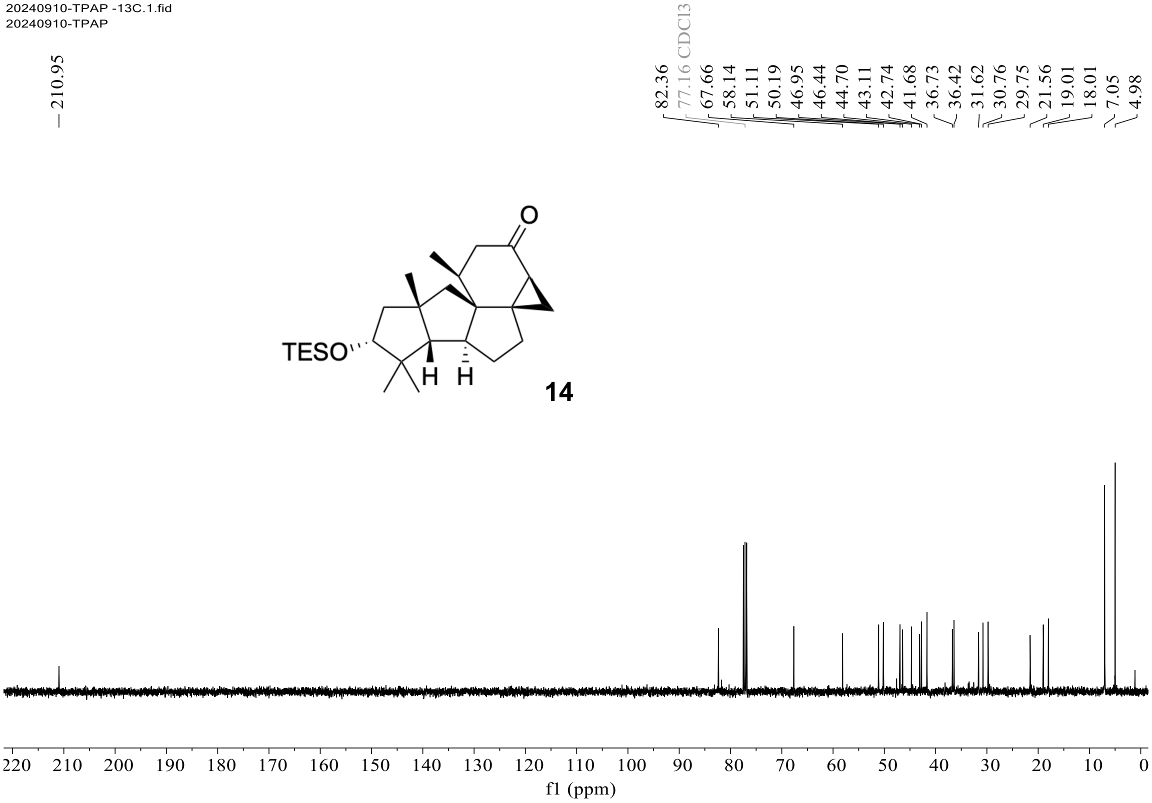

^{1}H (400 MHz) and $^{13}C\{^{1}H\}$ (100 MHz) NMR spectra of **15** in $CDCl_3$

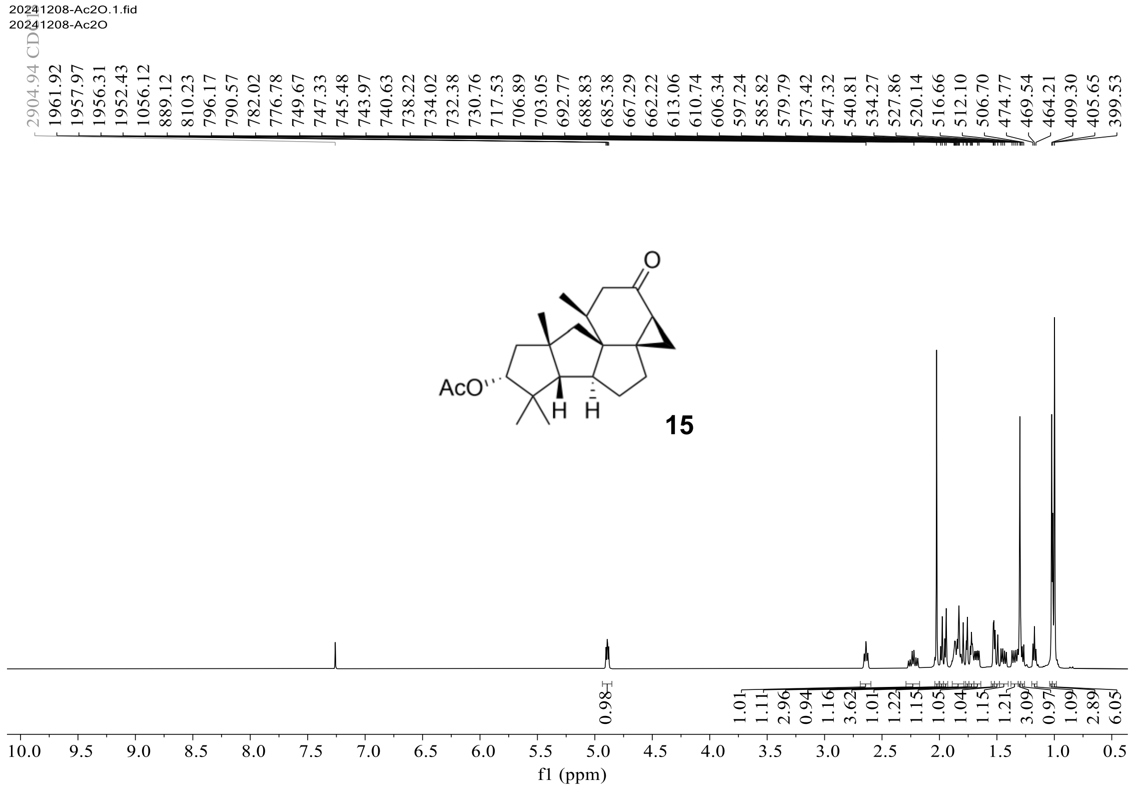

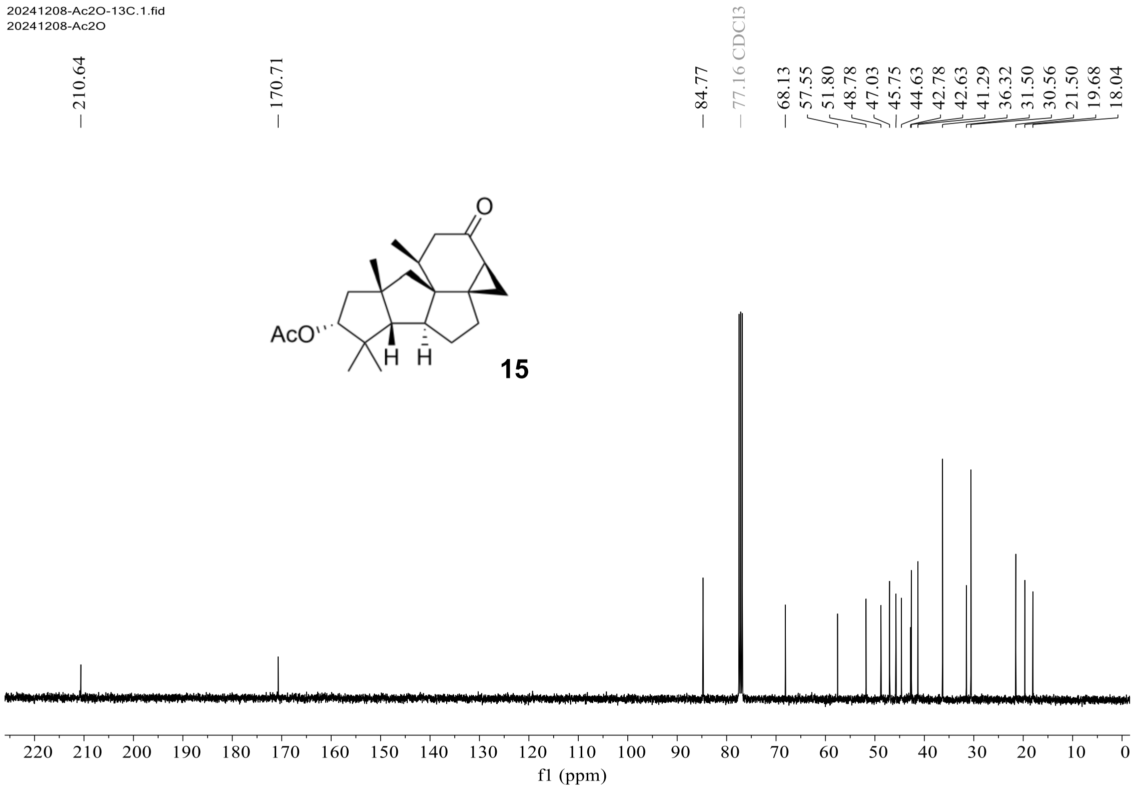

^{1}H (400 MHz) and $^{13}C\{^{1}H\}$ (100 MHz) NMR spectra of **16** in $CDCl_3$

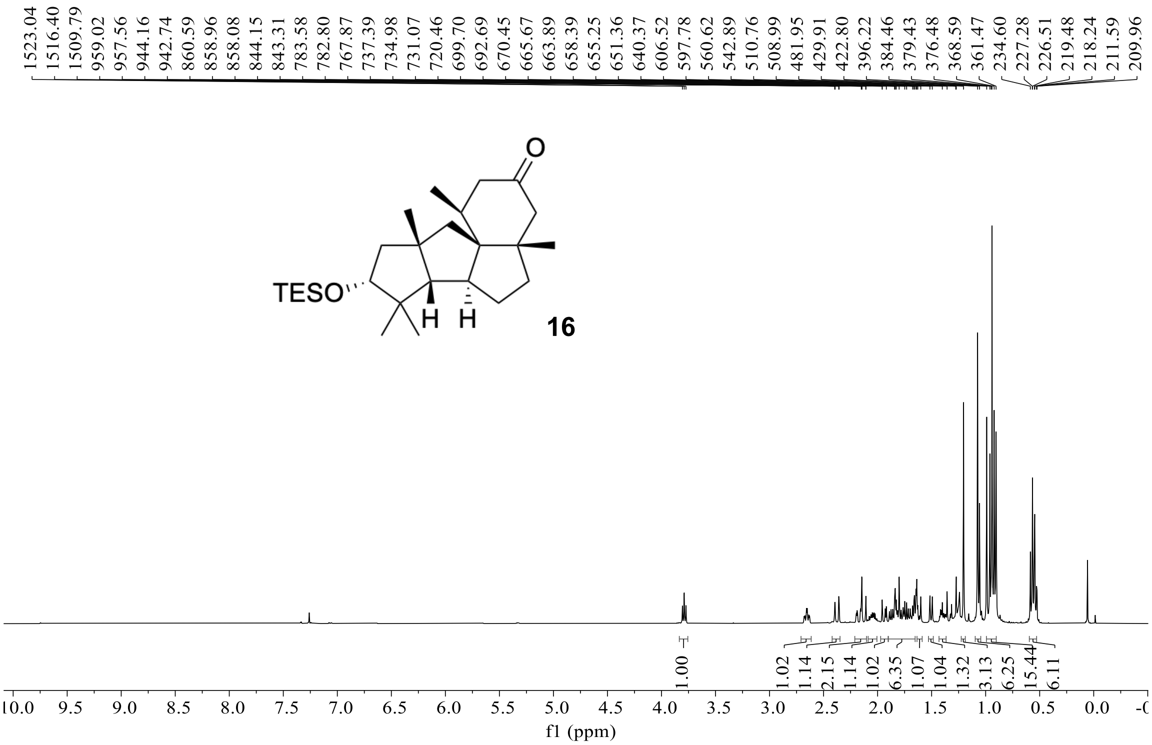

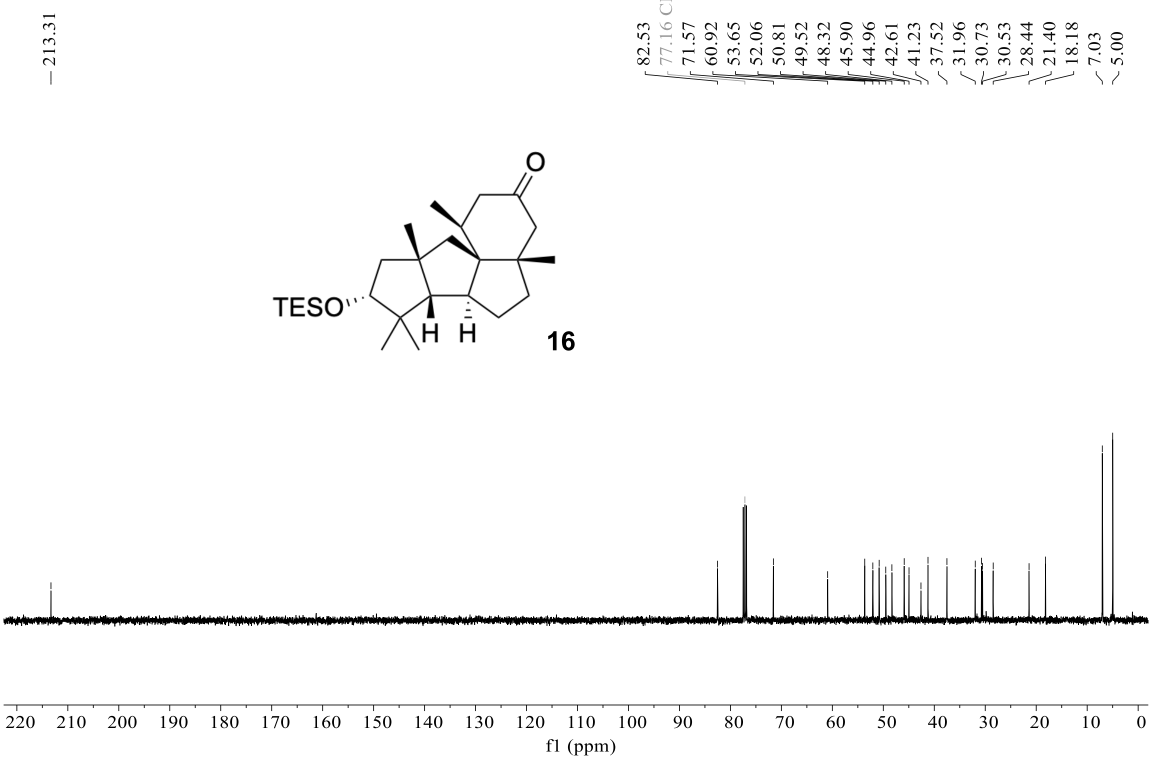

^1H (400 MHz) and $^{13}C\{^1H\}$ (100 MHz) NMR spectrum of **17** (rr = 6:1) in $CDCl_3$

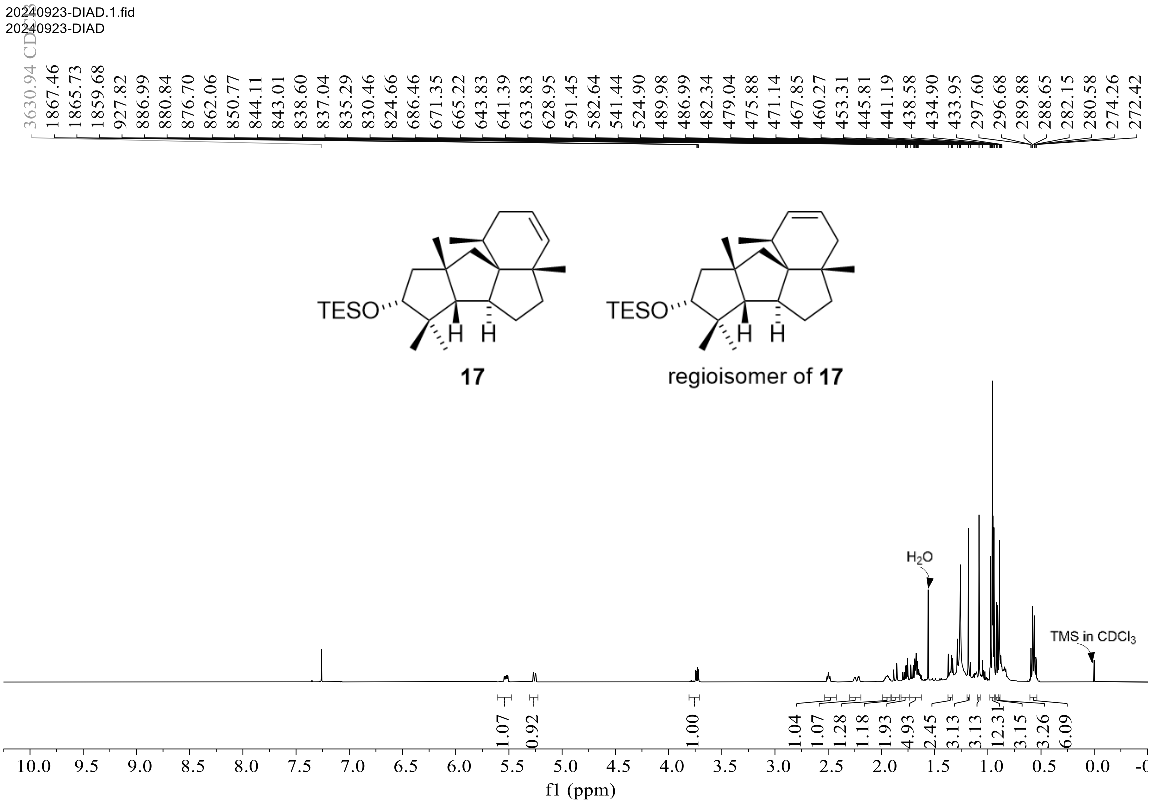

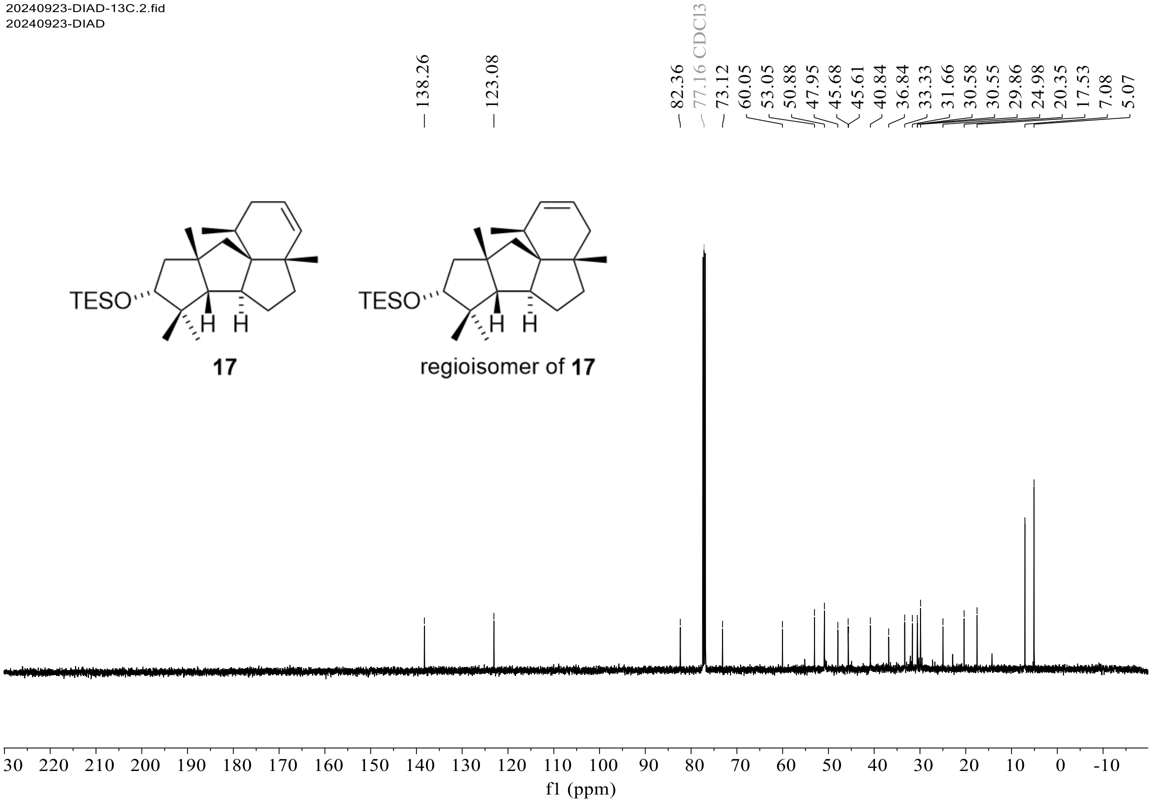

^{1}H (400 MHz) and $^{13}C\{^{1}H\}$ (100 MHz) NMR spectra of leptosphin C (**1**) in $CDCl_3$

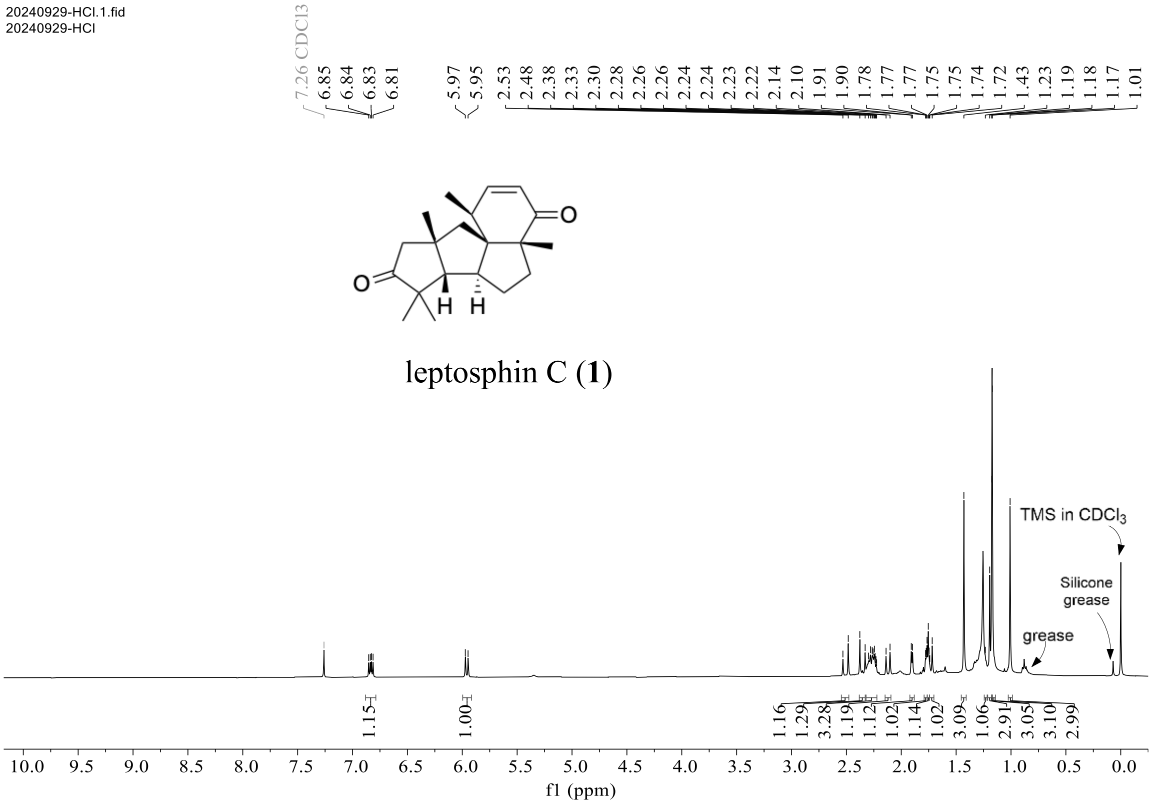

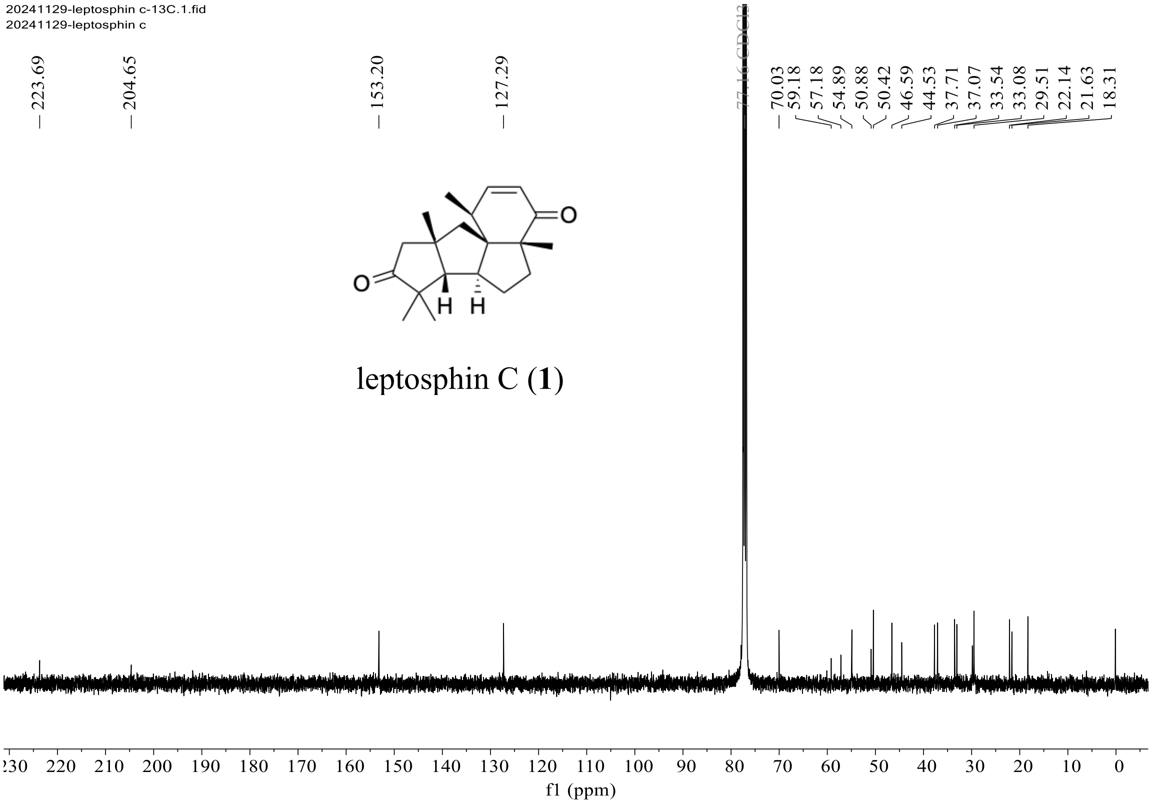

VIII. References

1. Pasha, M. A.; Krishna, A. V.; Ashok, E.; Ramachary, D. B. Organocatalytic Reductive Propargylation: Scope and Applications. *J. Org. Chem.* **2019**, *84*, 15399-15416.

2. Wen, S.; Li, P.; Wu, H.; Yu, F.; Liang, X.; Ye, J. Enantioselective organocatalytic phospha-Michael reaction of α,β-unsaturated ketones. *Chem. Commun.* **2010**, *46*, 4806-4808.

3. Zhao, Y.; Truhlar, D. G. The M06 suite of density functionals for main group thermochemistry, thermochemical kinetics, noncovalent interactions, excited states, and transition elements: two new functionals and systematic testing of four M06-class functionals and 12 other functionals. *Theor. Chem. Acc.* **2008**, *120*, 215-241.

4. Petersson, G. A.; Bennett, A.; Tensfeldt, T. G.; Al-Laham, M. A.; Shirley, W. A.; Mantzaris, J. A complete basis set model chemistry. I. The total energies of closed-shell atoms and hydrides of the first-row ele-ments. *J. Chem. Phys.***1988**, *89*, 2193-2218.

5. Weigend, F.; Ahlrichs, R. Balanced Basis Sets of Split Valence, Triple Zeta Valence and Quadruple Zeta Valence Quality for H to Rn: Design and Assessment of Accuracy. *Phys. Chem. Chem. Phys*. **2005**, *7*, 3297-3305.

6. Weigend, F. Accurate Coulomb-Fitting Basis Sets for H to Rn. *Phys. Chem. Chem. Phys.* **2006**, *8*, 1057-1065.

7. Miertus, S.; Scrocco, E.; Tomasi, J. Electrostatic interaction of a solute with a continuum. A direct utilization of AB initio molecular poten-tials for the prevision of solvent effects. *Chem. Phys.* **1981**, *55*, 117-129.

8. Frisch, M. J.; Trucks, G. W.; Schlegel, H. B.; Scuseria, G. E.; Robb, M. A.; Cheeseman, J. R.; Scalmani, G.; Barone, V.; Petersson, G. A.; Nakatsuji, H.; Li, X.; Caricato, M.; Marenich, A. V.; Bloino, J.; Janesko, B. G.; Gom-perts, R.; Mennucci, B.; Hratchian, H. P.; Ortiz, J. V.; Izmaylov, A. F.; Sonnenberg, J. L.; Williams-Young, D.; Ding, F.; Lipparini, F.; Egidi, F.; Goings, J.; Peng, B.; Petrone, A.; Henderson, T.; Ranasinghe, D.; Zakrzewski, V. G.; Gao, J.; Rega, N.; Zheng, G.; Liang, W.; Hada, M.; Eha-ra, M.; Toyota, K.; Fukuda, R.; Hasegawa, J.; Ishida, M.; Nakajima, T.; Honda, Y.; Kitao, O.; Nakai, H.; Vreven, T.; Throssell, K.; Montgomery, J. A., Jr.; Peralta, J. E.; Ogliaro, F.; Bearpark, M. J.; Heyd, J. J.; Brothers, E. N.; Kudin, K. N.; Staroverov, V. N.; Keith, T. A.; Kobayashi, R.; Nor-mand, J.; Raghavachari, K.; Rendell, A. P.; Burant, J. C.; Iyengar, S. S.; Tomasi, J.; Cossi, M.; Millam, J. M.; Klene, M.; Adamo, C.; Cammi, R.; Ochterski, J. W.; Martin, R. L.; Morokuma, K.; Farkas, O.; Foresman, J. B.; Fox, D. J. *Gaussian 16 Rev. C.01*, Gaussian Inc.: Wallingford, CT, 2016.
